# Factors that shape recurrent miscarriage care experiences: findings from a national survey

**DOI:** 10.1186/s12913-023-09347-1

**Published:** 2023-03-31

**Authors:** Caragh Flannery, Marita Hennessy, Rebecca Dennehy, Karen Matvienko-Sikar, Con Lucey, Jennifer Ui Dhubhgain, Keelin O’Donoghue

**Affiliations:** 1 Pregnancy Loss Research Group, Department of Obstetrics & Gynaecology, University College Cork, Cork, Ireland; 2 INFANT Research Centre, University College Cork, Cork, Ireland; 3 School of Public Health, University College Cork, Cork, Ireland; 4 RE:CURRENT Research Advisory Group, Pregnancy Loss Research Group, Department of Obstetrics & Gynaecology, University College Cork, Cork, Ireland

**Keywords:** Recurrent miscarriage, Pregnancy loss, Patient-centred care, Patient experience, Care quality

## Abstract

**Background:**

Learning what matters to women/couples with recurrent miscarriage (RM) is essential to inform service improvement efforts and future RM care practices. Previous national and international surveys have examined inpatient stays, maternity care, and care experiences around pregnancy loss, but there is little focus on RM care. We aimed to explore the experiences of women and men who have received RM care and identify patient-centred care items linked to overall RM care experience.

**Methods:**

Between September and November 2021, we invited people who had experienced two or more consecutive first trimester miscarriages and received care for RM in Ireland in the ten-year period prior to participate in a cross-sectional web-based national survey. The survey was purposefully designed and administered via Qualtrics. It included questions on sociodemographics, pregnancy and pregnancy loss history, investigation and treatment for RM, overall RM care experience, and patient-centred care items at various stages of the RM care pathway such as respect for patients' preferences, information and support, the environment, and involvement of partners/family. We analysed data using Stata.

**Results:**

We included 139 participants (97% women, *n* = 135) in our analysis. Of the 135 women, 79% were aged 35–44 years (*n* = 106), 24% rated their overall RM care experience as poor (*n* = 32), 36% said the care they received was much worse than expected (*n* = 48), and 60% stated health care professionals in different places did not work well together (*n* = 81). Women were more likely to rate a good care experience if they had a healthcare professional to talk to about their worries/fears for RM investigations (RRR 6.11 [95% CI: 1.41–26.41]), received a treatment plan (*n* = 70) (RRR 3.71 [95% CI: 1.28–10.71]), and received answers they could understand in a subsequent pregnancy (*n* = 97) (RRR 8 [95% CI: 0.95–67.13]).

**Conclusions:**

While overall experience of RM care was poor, we identified areas that could potentially improve people’s RM care experiences – which have international relevance – such as information provision, supportive care, communication between healthcare professionals and people with RM, and care coordination between healthcare professionals across care settings.

**Supplementary Information:**

The online version contains supplementary material available at 10.1186/s12913-023-09347-1.

## Background

Recurrent miscarriage (RM) affects 1–3% of the reproductive-aged population [1]. However, international guidelines vary in how it is defined [2]. The Royal College of Obstetricians and Gynaecologists [3] define RM as three or more first-trimester miscarriages (which do not have to be consecutive or with the same partner). The Practice Committee of the American Society for Reproductive Medicine [4] describes it as the loss of two or more consecutive pregnancies. Similarly, the European Society of Human Reproduction and Embryology now also define RM as the loss of two or more pregnancies, with the sequence of the miscarriages not necessarily consecutive [5].

Guidelines recommend that care for women with RM should be offered within a dedicated RM clinic [6, 7]. Healthcare professionals with the necessary skills and expertise [5, 8, 9], and those with a sub-specialisation in RM, provide a better standard of care when assessed against current guidelines [10]. RM clinics are consultant-led, non-acute and offer specialist investigations, support, and treatment to women/couples [6, 7]. In addition, they provide care plans to reduce the risk of further losses through treatments and addressing modifiable risk factors, where appropriate [6, 7]. Ideally, couples are seen together and given accurate information to facilitate decision-making about future pregnancies [5]. While there is limited evidence that this approach improves pregnancy outcomes, couples report valuing such care plans, and guidelines advocate for this approach [1, 11].

Quality in healthcare is shaped by the experiences and engagement of patients, families, caregivers, and professionals [12–17]. While ensuring that patients' perspectives and contributions to their healthcare decisions are considered and responded to accordingly, in general, it is not perceived to occur between women and maternity services following RM [18, 19]. While clinical practice guidelines for RM describe investigations and treatments, some do not provide mechanisms to ensure patient-centred care is guided by the values and needs of women/couples [20, 21]. As patient experience is multifaceted [22], learning what matters to women/couples during RM is essential to inform service improvement efforts and future RM care practices.

Previous national and international surveys have examined inpatient stays, maternity care, and care experiences around pregnancy loss, but there is little focus on RM care [23–27]. This study aimed to explore the experiences of women and men who have received RM care in Ireland and identify patient-centred care items linked to overall RM care experience.

## Methods

The Strengthening the Reporting of Observational Studies in Epidemiology checklist [28] was used to inform reporting of the findings. Ethical approval was granted by the Clinical Research Ethics Committee of the Cork Teaching Hospitals, University College Cork (ECM 4 (jj) 09/03/2021 & ECM 3 (jj) 19/10/2021).

### Study design

A cross-sectional study using an anonymous web-based survey was conducted to examine the experience of people who have interacted with the health services following RM.

### Sample selection and survey distribution

Women and men over 18 who experienced two or more consecutive first-trimester miscarriages in the preceding ten years (2010–2021), and who received care for RM in Ireland were invited to participate. Two or more consecutive first-trimester miscarriages were selected based on growing consensus and updated guidelines [5, 29], and the period of ten years was chosen to reflect the research and investment in miscarriage services in Ireland following the Miscarriage Misdiagnosis Review in 2010 [30–32]. The terms women and men are used throughout the paper, but participants were asked to identify themselves as ‘Mother/I carried the pregnancy’ or ‘Father/partner’ in the survey.

We used self-selection or voluntary response sampling, a type of non-probability sampling, where individuals volunteer themselves, i.e. responded to an open call for participants. This is a common approach for samples that need to meet specific criteria. We did this to develop an understanding of a smaller, under researched population in Ireland. We distributed the survey through existing professional, collegial and support networks, and the Clinical Midwife Specialists in Bereavement and Loss in each of the 19 maternity units/hospitals across Ireland from September to November 2021. The link to the survey was shared through email distribution lists, websites, newsletters, and social media, including those of the research team and the INFANT Research Centre, the Pregnancy Loss Research Group, and miscarriage/ pregnancy loss organisations. Recruitment materials, including posters/flyers and business cards, were tailored to women and men by including photos and quotes from those with lived experience of RM, which were also distributed nationally throughout pregnancy loss clinics and early pregnancy assessment units. Women and men with recurrent miscarriage in line with the eligibility criteria were invited to take part in the study through this open call for participants. Information about the study was presented to potential participants when they clicked the survey link and they then provided informed consent prior to completing the survey.

### Survey design

In a questionnaire consisting of 10 sections, 165 questions were purposefully designed using relevant literature [1, 19, 33, 34] and existing care experience surveys [5, 12, 27, 35] related to maternity care and/or pregnancy loss. Research findings from the RE:CURRENT Project [2, 36] informed the questionnaire, specifically qualitative research with healthcare professionals and women and men who had experienced RM [37] to tailor care experience questions to this cohort and ensure that relevant questions were being asked. The final questionnaire (see Additional File 1) was developed by the Research Team in consultation with key stakeholders, including parent advocates from the RE:CURRENT Research Advisory Group.

The structured questionnaire consisted of questions across several areas, including sociodemographic information, pregnancy and pregnancy loss history, investigations, receiving results and treatment for RM, follow-up care for subsequent pregnancies, the impact of RM, information, and support provision. The RE:CURRENT Project research team, and members of the RE:CURRENT Research Advisory Group, and Pregnancy Loss Research Group at Cork University Maternity Hospital, piloted the survey. After this pilot, changes were made to the wording, layout, and the selection of required responses within the online survey platform. The survey was formatted and managed using Qualtrics [38] with the advice and support of the RE:CURRENT Research Advisory Group.

### Survey measures

#### Stages of the RM care pathway

Tailored questions for women and men were used, with women asked more specific questions relating to the care they received to explore their experiences at various stages of the RM pathway.

For investigations, women were asked if they had investigations for RM (*yes vs no*); if investigations were offered (*vs requested*); the wait time for investigations (*less than one month, 1–2 months, 3–4 months, 5–12 months, I don't know/I can't remember*); types of medical tests (*yes* vs *no*) for medical history, blood test, ultrasound, MRI, hysteroscopy, genetic testing of pregnancy tissue, genetic tests or other. Finally, women were asked if they felt that their healthcare professionals did everything to investigate the cause of their RM (*no, yes definitely, yes to some extent*).

Women were asked if they received their results (*yes vs no*); the wait time for results (*less than one month, 1–2 months, 3–4 months, 5–12 months, I don't know/I can't remember*); who provided their results (*yes vs no*) general practitioner, midwife or nurse in the hospital, a sonographer, consultant in a public hospital, doctor in a public hospital, private consultant, healthcare professional at a fertility clinic, admin staff or other; if results were received by (*phone, email, face-to-face, virtual contact, other*) and if the results provided answers for the cause of their RM (*no, yes, I don't know*). Women were also asked if a treatment plan was put in place (*yes vs no*); if their healthcare professional did everything they could treat their RM (*no, yes definitely, yes to some extent*); if they had a subsequent pregnancy (*yes vs no*); if they were offered early reassurance scans (*yes vs no*) and if their healthcare professionals did everything to support them during their subsequent pregnancy (*no, yes definitely, yes to some extent*).

#### Patient-centred care items

The survey contained additional patient-centred care items [33], including questions relating to respect for patients' preferences, information and support, the environment and involvement of family at various stages of the RM care pathway (Table 1).

**Table 1:** Patient-centred care items included in the care experience survey

*Patient-Centred Care Items*^*a*^	*Care experience survey items and responses*
*Care coordination*	-Enough time to discuss [*No, yes]* -Involved in decisions [*No, yes*] -Treated with respect and dignity [*No, yes always, yes sometimes*] -Confidence and trust in HCP [*No, yes always, yes sometimes*] -One HCP said one thing, another said something different [*often, sometimes, only once, never*] -HCP deliberately not telling them something [*often, sometimes, only once never*]^b^ -Staff in different places work well together [*No, yes definitely, yes to some extent*]
*Information & support*	-Given enough information [*No, yes*]^c^ -Given written information [*No, yes*]^c^ -If questions, did you get answers that you could understand [*No, yes always, yes sometimes, I did not have the opportunity to ask questions, I did not need to ask questions*] -Who to contact if you had questions/concerns? [*No, yes]* -HCP talk to about your worries and fears [*No, yes*]
*Environment*	-Rate the waiting area(s) & consultation area [*Good, satisfactory, poor*]
*Involvement of family & friends*	-Did anyone attend the appointments [*No, yes, not facilitated due to covid*]^d^ -Partner wanted to talk to a HCP [*No, yes, I did not have a partner*]

^a^Questions were asked for each stage of the RM care pathway (investigations, receiving results, treatment plan, subsequent pregnancy); ^b^recoded to Yes/No; ^c^not asked for subsequent pregnancy; ^d^not asked for treatment plan

#### Outcome measure

Women and men were asked to rate their overall RM care experience, on a scale from 1–10, with one being 'a very poor experience' and ten 'a very good experience'. Due to small numbers in some categories, participant ratings were recoded as *poor* (rating 1–3), *satisfactory* (rating 4–6) and *good* (rating 7–10) for this analysis.

#### Participant characteristics

Women and men were asked to provide details of their: age (*18–24 years, 24–34 years, 35–44 years, 55–64 years, 65* + *years*); nationality (*Irish vs. non-Irish*); relationship status (*married, living with a partner, separated or divorced, single, prefer not to say*); education (*primary school or less, some secondary school, completed secondary school, post-secondary school technical training, university degree, postgraduate certificate or diploma, postgraduate degree (Masters or PhD)*); employment (*employed full-time, employed part-time, self-employed, employed casually, full-time student, part-time student, not employed, prefer not to say, other*); medical cover (*medical card or GP visit cardholder, private health insurance, none of the above*); number of consecutive losses (*two consecutive, three consecutive, more than three consecutive*); the year initial RM care received (*between 2011—2021*); ever diagnosed with infertility (*yes vs no*).

### Analysis

The data were checked by CF to ensure all participants met the inclusion criteria. Analysis was carried out using Stata V.13 [39]. Descriptive analyses were carried out for all variables through the RM care pathway. Associations between sample characteristics and care experience were explored using χ2 test. Unadjusted multinomial logistic regression was conducted to examine the association between patient-centred care items throughout the RM care pathway and overall RM care experience rating. Adjusted multinomial logistic regression analysis was not performed due to varying sample sizes through the RM pathway.

## Results

### Sample characteristics

A total of 213 participants completed the survey. However, 74 participants were not eligible for this analysis as they did not experience a consecutive miscarriage (*n* = 63) or did not receive care between 2011 and 2021 (*n* = 11). Of the eligible participants (*n* = 139), 97% were women (*n* = 135). Due to the small number of men (*n* = 4) (see Additional File 2), this section only presents results for women who participated. Of the women participants, 79% were aged 35–44 years (*n* = 106), 85% were married (*n* = 114), 39% had postgraduate degrees (*n* = 53) with 80% having private health insurance (*n* = 108). Furthermore, 57% had experienced two consecutive miscarriages (*n* = 77), 75% had received care in the last five years (2016–2021), and 24% had been diagnosed with infertility (*n* = 32) (Table 2).

**Table 2:** Participant characteristics

*Variable (n* = *135)*	*N (%)*
***Age category***	
* 24–34 years*	23 (17.04)
* 35–44 years*	106 (78.52)
* 55–64 years*	6 (4.44)
***Cultural***	
* White – Irish*	128 (94.81)
* Other white background*	6 (4.44)
* Asian or Asian Irish*	1 (0.74)
***Nationality***	
* Irish*	128 (94.81)
* Other*	7 (5.19)
***Relationship status***	
* Married*	114 (84.44)
* Living with a partner*	16 (11.85)
* Separated or divorced*	1 (0.74)
* Single*	3 (2.22)
* Prefer not to say*	1 (0.74)
***Education***	
* Secondary school or less*	6 (4.44)
* Post-secondary school technical training*	9 (6.67)
* University degree*	36 (26.67)
* Postgraduate Certificate or Diploma*	31 (22.96)
* Postgraduate Degree (Masters or PhD)*	53 (39.26)
***Employment***	
* Employed full-time*	104 (77.04)
* Employed part-time*	14 (10.37)
* Self-employed*	5 (3.70)
* Full-time student*	1 (0.74)
* Part-time student*	3 (2.22)
* Not employed*	4 (2.96)
* Prefer not to say*	1 (0.74)
* Other*	3 (2.22)
***Medical cover***	
* A medical or GP visit card holder*	8 (5.93)
* Private health insurance holder*	108 (80.00)
* None of the above*	19 (14.07)
***Consecutive loss***	
* Two*	77 (57.04)
* Three*	34 (25.19)
* Four or more*	24 (17.79)
***Year received RM care***	
* 2011–2015*	34 (25.19)
* 2016–2021*	101 (74.81)
***Diagnosed with infertility***	
* No*	103 (76.30)
* Yes*	32 (23.70)

### Participant's rating of overall RM care experience

Rating of overall RM care experience was characterised as follows: poor (44%, *n* = 60), satisfactory (39%, *n* = 52) and good (17%, *n* = 23). Based on χ2 test, overall RM care experience rating was not associated with participant characteristics (Table S3.1, Additional File 3).

### Participant's experiences of the various stages of the RM care pathway

**Fig. 1:**
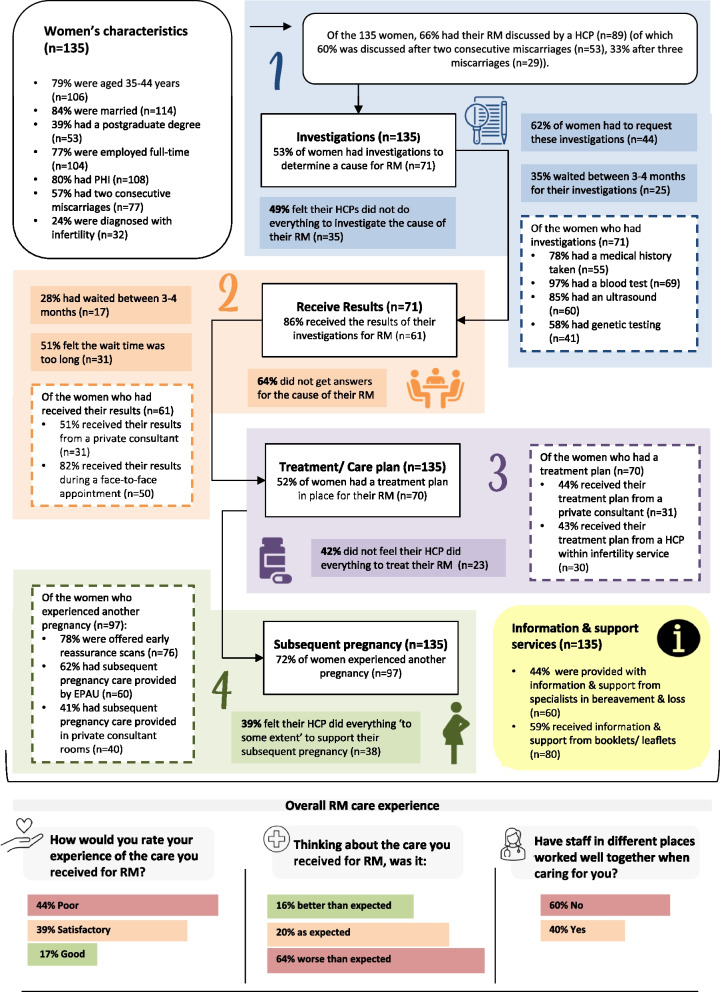
Overview of women’s experiences of the various stages of the RM care pathway *HCP, healthcare professional; RM, recurrent miscarriage; EPAU, early pregnancy assessment unit; PHI, private health insurance* ​

Participants' experiences of the various stages of the RM care pathway are depicted in Fig. 1 (Also, see supporting data, Tables S3.2-S3.7, Additional File 3). Of the participants (*n* = 135), 66% had discussed RM with a healthcare professional, 53% had investigations for RM (*n* = 71), 62% requested these investigations (*n* = 44), 35% waited between 3–4 months for investigations (*n* = 25) to take place, and 49% felt their healthcare professional did not do everything to investigate their RM (*n* = 35).

Of the participants who had investigations (*n* = 71), 86% received their results (*n* = 61), 28% waited 3–4 months for results (*n* = 17), 51% felt the wait time was too long, 51% received results from a private consultant (*n* = 31), and 64% did not get answers about causes of their RM (*n* = 39). Furthermore, 52% had a treatment plan in place (*n* = 70), of which 44% received their treatment plan from a private consultant (*n* = 31), and 42% did not feel their healthcare professional did everything to treat their RM (*n* = 23).

Finally, 72% experienced another pregnancy (*n* = 97), of which 78% were offered early reassurance scans (*n* = 76), and 39% felt their healthcare professional 'to some extent' did everything to support their subsequent pregnancy (*n* = 38). When thinking about their RM care, 64% said it was worse than expected (*n* = 87), with 60% stating that healthcare professionals did not work well together during their care (*n* = 84).

### Patient-centred care items throughout the RM care pathway and overall RM care experience

In the unadjusted multinomial logistic regression, some patient-centred care items for investigations, receiving results, treatment plans and subsequent pregnancy were linked to either satisfactory or good RM care experience (see Additional File 4).

For participants who had RM investigations (*n* = 71), those who requested these were less likely to report a good RM care experience relative to those who were offered investigations (RRR 0.19 [95% CI:0.48–0.71]). Participants who received verbal information (RRR 13.10 [95% CI: 2.48–60.30]), written information (RRR 5.37 [95% CI: 1.06–27.00]) and answers they could understand regarding investigations (RRR 8.89 [95% CI: 1.03–76.68]) were more likely to report a good overall RM care experience. Also, participants who had a healthcare professional to talk to about their worries/fears (RRR 6.11 [95% CI: 1.41–26.41]) and whose partner could ask questions about investigations (RRR 3.83 [95% CI: 1.01–14.48]) were more likely to report a good overall RM care experience.

For participants who received their RM investigation results (n = 61), those who received information about their results (RRR 10.21 [95% CI: 1.15–90.53]) had a healthcare professional to talk to about their worries/fears (RRR 18.70 [95% CI: 2.09–167.27]) and whose partner could ask questions about their investigation results (RRR 5.88 [95% CI: 1.30–26.51]) were more likely to report a good overall RM care experience.

Participants who received a treatment plan (*n* = 70) were more likely to rate a good care experience (RRR 3.71 [95% CI: 1.28–10.71]). Also, participants that had a healthcare professional to talk to about their worries and fears surrounding their treatment (RRR 12 [95% CI: 2.25–63.98]) and who reported that they felt their healthcare professional did everything to treat their RM (RRR 36 [95% CI: 4.05–320.12]) were more likely to rate a good care experience.

For participants who had a subsequent pregnancy (*n* = 97), those who were offered reassurance scans were more likely to rate a satisfactory care experience (RRR 3.27 [95% CI: 1.10–9.67]) or a good care experience (vs poor) (RRR 8.96 [95% CI: 1.07–74.91]) compared to those who did not get reassurance scans. Participants who received answers they could understand (RRR 8 [95% CI: 0.95–67.13]), had someone they could contact with questions (RRR 12.00 [95% CI: 2.40–60.05]) and had a healthcare professional they could talk to about their worries/fears (RRR 6.43 [95% CI: 1.29–32.0]) concerning their subsequent pregnancy were more likely to rate a good care experience.

## Discussion

This study aimed to explore the experiences of women and men who have received RM care in the Republic of Ireland, exploring their experiences at various stages of the RM care pathway and identifying patient-centred care items linked to their overall care experience. Despite an open call to recruit women and men, only four men participated in this survey, highlighting the already recognised challenge of recruiting men for reproductive health and pregnancy loss research [40, 41]. Previous research indicated that men may 'feel in the way' during the miscarriage process; therefore, more inclusivity is needed, and a couple-focused approach to care and support implemented [42, 43].

Of the women who participated (*n* = 135), 44% rated a poor overall RM care experience (*n* = 60), with 64% stating the care was worse than expected (*n* = 87) and 60% reporting that healthcare professionals did not work well together (*n* = 84) then providing their RM care. However, across the RM care pathway, a range of patient-centred care items such as having a healthcare professional to talk to about worries and fears, partners involvement, receiving enough information and having time to discuss and be involved in decisions regarding investigations and subsequent pregnancies were linked to a satisfactory or good rating of RM care experience.

A clear gap exists between the care women want and the care they receive [19, 37, 44]. Like previous international research, women reported a poor RM care experience, with RM care worse than expected, citing a lack of information, communication, and follow-up care [45–48]. In Ireland, miscarriage is not officially recorded and is most likely underreported, as not all women attend hospital for miscarriage care [16–19]. Despite increased allocation of resources following the Miscarriage Misdiagnosis Review in 2010 in Ireland, pregnancy loss/miscarriage services are still seen as a low priority [30, 31]. Miscarriage data is required to compare miscarriage/RM rates among countries, accelerate research, improve patient care, and support advocacy efforts and policy development [1].

In this study, women felt that healthcare professionals across different care settings did not work well together, indicating a lack of communication, undermining RM care and support consistency. Multidisciplinary teams are required for RM to enhance and encourage knowledge-sharing between healthcare professionals, allowing for effective communication between primary and secondary care and other services (emergency department/fertility/perinatal mental health). Moreover, in line with previous international research, results indicated a better care experience if women were given time to discuss and actively contribute to their RM care [44, 49]. Women want more effective doctor-patient communication, which requires interventions to change long-established behaviours and perceptions of both staff and patients [47]. Providing care through dedicated RM clinics would facilitate a multi-disciplinary approach, open communication channels, and encourage teamwork between healthcare professionals.

Research indicates that providing physical, emotional and psychological support, and information and education for family and friends about pregnancy loss is key to improving care experience [24]. Women were more likely to rate a satisfactory or good RM care experience if they had received enough information regarding their miscarriage, particularly concerning investigations and results. The consequences of RM can be profound and life-changing for women/partners/families and, as such, the provision of supportive care should be central to the management of women/couples [45]. In tandem with emotional and informational support from healthcare professionals, as varying consistency/trustworthiness of information exists, women and men should be supported to mobilise evidence-based information and support for themselves [46]. Geller and colleagues [46] provided a table of well-established websites that can be distributed to women and valuable educational resources for healthcare professionals. Women are more likely to rate a satisfactory or good RM care experience if they had a healthcare professional to talk to about their worries and fear at each stage of the RM care pathway. In a study where women who attended follow-up appointments with healthcare professionals to discuss their miscarriages, women were less likely to experience psychological distress [48].

Overall, these findings align with the work of others internationally that has highlighted the need to invest in a better model of care that supports women/couples with RM while including women/couples in improvement efforts [12, 50, 51]. Dedicated RM clinics, where skilled and experienced healthcare professionals, provide women/couples with treatment plans, education, and continuous support into the next pregnancy within a dedicated service have been implemented nationally and internationally [7] as a potential solution. In the UK, a graded model of care has been put forward to address the balance between evidence-based management and supportive care and healthcare resources, with care pathways based on the first and subsequent miscarriages [11]. To ensure patient-centred care items are achieved, these approaches offer concrete solutions to help individualise care according to women’s and their partner's needs and preferences [6, 7]. Future research in needed to check the scalability and sustainability of such models of care to maximise the impact on health outcomes and to respond to budgetary constraints in the health system [52, 53].

### Strengths and limitations

Our findings add to the extant knowledge base on the care experiences of people who experience pregnancy loss, addressing an important gap in the literature specifically regarding RM. While our study was conducted in Ireland, our findings regarding overall care experiences are similar to those observed in other international studies. This study employed a strict inclusion criterion that included women/men who had experienced two or more first-trimester miscarriages, and as the guidelines and definitions for RM vary, some women/men were excluded from this analysis. As ten years were chosen to reflect the research and investment in miscarriage services in Ireland since 2010 following the Miscarriage Misdiagnosis Review [30], recall bias may be present. Although less is known about paternal recall, previous studies on reproductive events have demonstrated that maternal recall has acceptably high reliability and is little affected by time from the event [54]. Many participants who took part in this study were of White Irish ethnicity. A more diverse sample in terms of ethnicity and socioeconomic background may have provided broader insight into the experience of RM care. As noted earlier, further work is also needed to engage men in this type of research and to elicit their views and experiences. As the survey was self-selecting, findings cannot be generalised to the Irish population. Most participants were older, of a higher socioeconomic status and well-educated, with private health insurance. Data on miscarriage is not routinely recorded in Ireland; however, cohort studies show that women with recurrent miscarriage tend to be older (aged ≥ 35 years) [55, 56]; age is a key demographic risk factor for miscarriage [1]. Our use of voluntary response sampling introduces some biases in the sample as some people are inherently more likely to volunteer than others, and our sample is not representative of the population under study. For example, negative bias can influence motivation to complete a task, women with negative experiences may be over-represented [57, 58]. Several variables were re-categorised for the analysis, easy interpretation, and presentation of results [59]. Each stage of the RM pathway had different sample sizes resulting in wide confidence intervals indicating the data does not provide a precise representation. While using statistical inference is not recommended in non-probability sampling, in this instance the confidence interval/*p*-value confronts the sample with a certain probability model, which enabled this analysis to highlight patient centred care items that predict/ influence overall care experience as data is limited in Ireland for RM. Therefore, results should be interpreted with caution.

Nonetheless, the care experience survey was a valuable tool for assessing RM care delivery processes and including women's experiences in quality improvement research [33]. Addressing an identified research gap, our results highlight patient centred care items that influence overall care experience ratings, and provide a starting point for future work. Building on previous surveys and input from the RE:CURRENT Research Advisory Group in the development, distribution and analysis added substantial strength to this study and has resulted in a tool that could be used/adapted in future research.

## Conclusions

This study provides an overview of women's experiences through the RM care pathway and identifies patient-centred care items that shape the overall rating of RM care experience. While overall experience of RM care was poor, areas that could potentially improve the care experience, included information provision, supportive care and communication between healthcare professionals and patients which reiterates the work of others [12, 45, 50, 51]. To ensure patient-centred care items are achieved, approaches such as RM clinics and graded approaches to providing RM care could be implemented globally to provide collaborative teamwork and a unified, holistic approach to RM care [11]. Overall, the results provide a better understanding of the drivers shaping care experiences to help inform and improve RM care.

## Supplementary Information

**Additional file 1. **RE:CURRENT Care Experience Survey.

**Additional file 2: Table S2.1.** Men’s characteristics. **Figure S2.1.** Location where the majority of RM investigations were carried out (Men)*.* **Figure S2.2.** Attendance at RM care appointments (Men). **Table S2.2.** Patient-centred care items for investigation, receiving results, treatment/plan of care, and subsequent pregnancy (Men). **Figure S2.3.** RM care experience (Men)*.*

**Additional file 3: Table S3.1.** Women’s characteristics by overall RM care experience rating. **Table S3.2.** Care received by women during investigations for RM. **Table S3.3.** Care received by women when receiving the results of their investigations for RM. **Table S3.4. **Care received by women when getting their treatment plan for RM**. Table S3.5**. Care received by women for a subsequent pregnancy following RM**.** **Table S3.6. **Information and support services used by women for RM.** Table S3.7.** Women’s overall RM care experience rating.

**Additional file 4:** **Table S4.1.** Unadjusted associations for patient-centred care items during investigations by overall care experience rating. **Table S4.2.** Unadjusted associations for patient-centred care items when receiving results by overall care experience rating. **Table S4.3.** Unadjusted associations for patient-centred care items for treatment/plan of care by overall care experience rating. **Table S4.4.** Unadjusted associations for patient-centred care items during subsequent pregnancy care by overall care experience rating.

## Data Availability

All data relevant to the study are included in the article or uploaded as supplementary information.

## Abbreviation

RM: Recurrent miscarriage
