# Supplementary material for: Factors that shape recurrent miscarriage care experiences: findings from a national survey
Source: BMC Health Serv Res. 2023 Mar 31;23:317. doi: 10.1186/s12913-023-09347-1 (PMC10064661; doi:10.1186/s12913-023-09347-1)
Supplement: Supplementary file 1 — Additional file 1. RE:CURRENT Care Experience Survey. [file 12913_2023_9347_MOESM1_ESM.docx]

Additional File 1.

RE:CURRENT Care Experience Survey

Start of Block: Consent

**RE:CURRENT Recurrent Miscarriage: Evaluating Current Services**
**Care Experience Survey**
**What is this survey about?**
The RE:CURRENT Care Experience Survey is a nationwide survey asking women and men about their care for recurrent miscarriage. The RE:CURRENT Project Management Group based at the INFANT Research Centre, University College Cork (UCC) and the Pregnancy Loss Research Group at Cork University Maternity Hospital (CUMH) is conducting the survey to provide insight into the care provided to those who experience recurrent miscarriage and to identify the types of services and supports that may help at such a time. The results will be used to improve existing services and supports. The survey forms part of an ongoing evaluation of recurrent miscarriage services in the Republic of Ireland, a two-year programme of research funded by the Health Research Board (HRB).

**Who can take part?**

Women and men over 18 who have experienced two or more consecutive first trimester miscarriages and were cared for in the Republic of Ireland in the last ten years are invited to complete the survey. The survey is available in English only.

 **What does participation in this study involve?**
 You will be asked to complete a survey about the care you received for recurrent miscarriage. The survey will take approximately 45-60 minutes to complete. You do not need to complete this survey in one sitting. If you leave the survey, the survey link will allow you to return to where you left off.

**Can I ask someone to help me fill in the survey?** Yes, you can ask someone to help you fill in the survey. You may also ask someone to fill in the survey on your behalf. However, please make sure that the answers given reflect your experience of care.

**What will happen to my information?**
UCC is the Data Controller for this project and owns the information collected. Your participation in this survey is anonymous, and all information will be treated confidentially. In keeping with policy, data will be retained for a minimum of ten years after the completion of the project. Anonymised results will be published in international peer-reviewed journals, presented at research meetings, and conferences and shared with key stakeholders involved in providing maternity services to inform service improvements.

**What are the risks and benefits of taking part in the survey?**
Taking part will give you a chance to share your experience of recurrent miscarriage care. You may not benefit personally; however, the survey results will help improve the quality of recurrent miscarriage services and supports in Ireland. We don’t envisage any negative consequences for you. Your participation will not affect your current or future care in any way. However, it is possible that thinking about your experience while completing the survey may cause distress. If this happens, please talk to your care provider or contact the support services below. These details will be provided again at the end of the survey.

[Pregnancy and Infant Loss Website](https://pregnancyandinfantloss.ie/)
[The Miscarriage Association of Ireland](http://www.miscarriage.ie/) provide peer to peer support to those who have experienced miscarriage
[Féileacáin](https://feileacain.ie/)provide peer to peer support to bereaved parents 
[A Little Lifetime](https://alittlelifetime.ie/) provide peer to peer support to bereaved parents
[Cork Miscarriage](http://www.corkmiscarriage.com) Information and Resources 

**Can I change my mind?**
Participation in the survey is voluntary and anonymous. You maintain the right to withdraw from the study up to the point of survey completion. If you decide to participate but then change your mind before finishing the survey, simply close your web browser. As the survey is completely anonymous, it will not be possible to identify your information to withdraw it after you complete the survey.

**Is this study approved by an ethics committee?**
Ethical approval for this study has been granted by the Clinical Research Ethics Committee of the Cork Teaching Hospitals (CREC). If you have further queries concerning your rights in connection with the research, you can contact CREC at Lancaster Hall, 6 Little Hanover Street, Cork or on 021-4901901/[crec@ucc.ie](http://crec@ucc.ie)

Once you have read this information, you are welcome to contact the research team if you have any questions regarding this study or if you have any concerns related to your involvement. **Please contact the research team:** Marita Hennessy, PhD,  Postdoctoral Researcher, [maritahennessy@ucc.ie](mailto:maritahennessy@ucc.ie), or Caragh Flannery, PhD, Postdoctoral Researcher, [cflannery@ucc.ie](mailto:cflannery@ucc.ie)

| Page Break |  |
| --- | --- |

**Consent Form**
**Declaration by Participant**
Please read each of the following statements carefully and indicate your agreement to participate below.
I have read the information above

I have had the opportunity to ask questions

I am an adult (over 18 years of age)

I understand the purposes, procedures, risks and benefits of taking part in this survey

I freely agree to participate in this research as described

Please click here to confirm that you have read the above and would like to take part

- Yes, I would like to participate (1)

**If you do not wish to participate, please close your web browser now.**

| Page Break |  |
| --- | --- |

We are very sorry that you and your family have experienced recurrent miscarriage. We understand that this is a difficult experience and hope that our research will help to improve future care. Your responses to this survey will help provide insight into the care provided to those who experience recurrent miscarriage and identify the types of services and supports that may help.

Sections within this survey will **relate to different aspects of your care** including investigations, receiving your results, treatment/plan of care and supportive services. You will be asked **similar questions in each section** which might seem repetitive but it will be useful for us to see what aspects of care are working well or not. 

**There are no right or wrong answers to the questions;** we would like to hear from you about your experiences. The survey should take about **45-60 minutes to complete,** but please take your time as you go through it.

End of Block: Consent

Start of Block: Main body

**Section 1: About you**
In this section, we will ask you some questions about you and your experience of pregnancy loss

How old are you today?

▼ 18-24 years (1) ... 65+ years (6)

| Page Break |  |
| --- | --- |

What is your cultural background?

- White - Irish (1)
- White - Irish Traveller (2)
- Other White background (3)
- Black or Black Irish - African (4)
- Black or Black Irish - Other Black background (5)
- Asian or Asian Irish - Chinese (6)
- Asian or Asian Irish - Other Asian background (7)
- Other, including mixed background. Please specify: (8) ________________________________________________

| Page Break |  |
| --- | --- |

What is your nationality?

- Irish (1)
- No nationality (2)
- Other. Please specify: (3) ________________________________________________

| Page Break |  |
| --- | --- |

What best describes your current relationship status?

- Married (1)
- Living with partner (2)
- In a relationship, but we are not living together (3)
- Separated or divorced (4)
- Single (5)
- Widowed (6)
- Prefer not to say (7)

| Page Break |  |
| --- | --- |

What is the highest level of education/training which you have completed to date?

- Primary school or less (1)
- Some secondary school (2)
- Completed secondary school (3)
- Post-secondary school technical training (4)
- University degree (5)
- Postgraduate Certificate or Diploma (6)
- Postgraduate Degree (Masters or PhD) (7)

| Page Break |  |
| --- | --- |

What best describes your current employment status? [If COVID-19 has affected your work, please indicate your usual employment status]

- Employed full-time (1)
- Employed part-time (2)
- Self-employed (3)
- Employed casually (4)
- Full-time student (5)
- Part-time student (6)
- Not employed (7)
- Prefer not to say (8)
- Other. Please specify: (9) ________________________________________________

| Page Break |  |
| --- | --- |

Which of the following best describes the type of health/medical cover that you currently hold?

- A medical card holder (1)
- A GP visit card holder (2)
- Private health insurance holder (3)
- Both a medical card and private health insurance holder (4)
- Both a GP visit care and private health insurance holder (6)
- None of the above (5)

| Page Break |  |
| --- | --- |

How many times have you/ your partner been pregnant and experienced pregnancy loss? [Please enter the number beside each of the following statements. Leave blank if 0]

|  | Number (1) |
| --- | --- |
| Living children (1) |  |
| First trimester miscarriage (before 12 weeks gestation) (2) |  |
| Second trimester miscarriage (12-24 weeks gestation) (3) |  |
| Ectopic pregnancy (4) |  |
| Stillbirth (my baby was born with no signs of life after 24 weeks gestation) (5) |  |
| Induced termination of pregnancy (6) |  |
| Neonatal death (my new-born baby died after birth and within 28 days of life) (7) |  |
| Infant death (my baby died in infancy / childhood, after 28 days of life) (8) |  |
| Other. Please specify: (9) |  |

| Page Break |  |
| --- | --- |

How many of you/ your partners first trimester miscarriages occurred consecutively (one after the other)?

▼ 0 (1) ... 10 (11)

Skip To: End of Survey If How many of you/ your partners first trimester miscarriages occurred consecutively (one after the... = 0

| Page Break |  |
| --- | --- |

What year did you first receive care for recurrent miscarriage?

▼ 2011 (1) ... I did not receive care between the years 2011-2021 (13)

| Page Break |  |
| --- | --- |

Have you ever been diagnosed with infertility (not pregnant after 12 months of trying) by a health professional?

- Yes (1)
- No (2)

| Page Break |  |
| --- | --- |

Have you/ your partner ever received treatment for a fertility issue?

- Yes, I have received treatment (1)
- Yes, my partner has received treatment (4)
- Yes, we both have received treatment (5)
- No (6)
- I don't know (7)

| Page Break |  |
| --- | --- |

Which best describes you?

- Mother / I carried the pregnancies (1)
- Partner / Father (2)

Skip To: End of Block If Which best describes you? = Partner / Father

| Page Break |  |
| --- | --- |

**Section 2: Investigations for Recurrent Miscarriage**
In this section, we will ask you about any investigations you may have had for recurrent miscarriage. We use the term **“investigations” to refer to any medical tests you and/or your partner might have undergone** to determine a cause for your recurrent miscarriage. If you have been investigated multiple times, we ask that you think about **the first time that you had investigations** for recurrent miscarriage.

Did a healthcare professional discuss your recurrent miscarriages with you?

- Yes (1)
- No (2)
- I don't know / I can't remember (3)

Skip To: Q21 If Did a healthcare professional discuss your recurrent miscarriages with you? = No

Skip To: Q21 If Did a healthcare professional discuss your recurrent miscarriages with you? = I don't know / I can't remember

| Page Break |  |
| --- | --- |

After how many miscarriages was recurrent miscarriage discussed with you? [Please enter the number of miscarriages below]

________________________________________________________________

| Page Break |  |
| --- | --- |

Who was the first healthcare professional to discuss recurrent miscarriage with you?

- GP (1)
- Midwife/Nurse in the hospital (2)
- Sonographer (healthcare professional who specialises in the use of ultrasound) (3)
- Consultant in a public hospital (4)
- Private Consultant (5)
- Doctor/midwife/nurse at a fertility clinic (6)
- Other. Please specify: (7) ________________________________________________
- I don't know / I can't remember (8)

| Page Break |  |
| --- | --- |

Have you had any investigations to try to find the cause of your recurrent miscarriage?

- Yes (1)
- No (2)
- I don't know/ I can't remember (3)

Skip To: Q89 If Have you had any investigations to try to find the cause of your recurrent miscarriage? = No

Skip To: Q57 If Have you had any investigations to try to find the cause of your recurrent miscarriage? = I don't know/ I can't remember

| Page Break |  |
| --- | --- |

Were you offered these investigations, or did you request them?

- I was offered investigation(s) (1)
- I requested investigation(s) (2)
- I don't know / I can't remember (3)

| Page Break |  |
| --- | --- |

After how many miscarriages were you referred for investigations for your recurrent miscarriage?

- 1 (1)
- 2 (2)
- 3 (3)
- 4 or more (4)
- I don't know/ I can't remember (5)

| Page Break |  |
| --- | --- |

How many years ago were you referred for investigation for recurrent miscarriage? [Please enter the number of years below]

________________________________________________________________

| Page Break |  |
| --- | --- |

Who was the first healthcare professional to refer you for investigation for your recurrent miscarriage?

- GP (1)
- Midwife/Nurse in the hospital (2)
- Sonographer (healthcare professional who specialises in the use of ultrasound) (3)
- Consultant in a public hospital (4)
- Private Consultant (5)
- Doctor/midwife/nurse at a fertility clinic (6)
- Other. Please specify: (7) ________________________________________________
- I don’t know/ I can’t remember (8)

| Page Break |  |
| --- | --- |

After referral, how long did you wait for an appointment for investigation(s) for your recurrent miscarriage?

- Less than one month (1)
- 1-2 months (2)
- 3-4 months (3)
- 5-6 months (4)
- 7-12 months (5)
- More than 12 months (7)
- I don't know / I can't remember (6)

| Page Break |  |
| --- | --- |

How did you feel about the length of time you waited from referral to your appointment for investigations?

- Too Short (1)
- About right (2)
- Too long (3)
- I didn't have to wait (4)
- I don't know / I can't remember (5)

| Page Break |  |
| --- | --- |

Did you take time off from work to attend your appointment for investigations?

- Yes. Please specify, on average, how many hours you took off from work (1) ________________________________________________
- No (2)
- I don't know / I can't remember (3)
- Not applicable (4)

Skip To: Q30 If Did you take time off from work to attend your appointment for investigations? = Not applicable

Skip To: Q30 If Did you take time off from work to attend your appointment for investigations? = No

Skip To: Q30 If Did you take time off from work to attend your appointment for investigations? = I don't know / I can't remember

| Page Break |  |
| --- | --- |

Was your job/work performance affected by attending your appointment for investigations?

- Missed a chance of promotion (1)
- Missed a deadline (2)
- No, it was not affected (3)
- I don’t know / I can't remember (4)
- Other. Please specify: (5) ________________________________________________

| Page Break |  |
| --- | --- |

Approximately how many appointments did you attend for these investigations overall? [Please select from drop-down list]

▼ 1 (1) ... I don't know/ I can't remember (10)

| Page Break |  |
| --- | --- |

Did you receive enough information about investigations for recurrent miscarriage?

- Yes, definitely (1)
- Yes, to some extent (2)
- No (3)
- I did not want or need this information (4)
- I don't know / I can't remember (5)

| Page Break |  |
| --- | --- |

Were you given any written information about investigations for recurrent miscarriage?

- Yes (1)
- No (2)
- I did not want or need any written or printed information (3)
- I don’t know / I can’t remember (4)

| Page Break |  |
| --- | --- |

Did you feel you had enough time to discuss investigations for recurrent miscarriage, and what they would involve?

- Yes, definitely (1)
- Yes, to some extent (2)
- No (3)
- I don't know / I can't remember (4)

| Page Break |  |
| --- | --- |

Were you involved as much as you wanted to be in decisions about investigations for recurrent miscarriage?

- Yes, definitley (1)
- Yes, to some extent (2)
- No (3)
- I don't know / I can't remember (4)

| Page Break |  |
| --- | --- |

When you had questions to ask about investigations for recurrent miscarriage, did you get answers that you could understand?

- Yes, always (1)
- Yes, sometimes (2)
- No (3)
- I did not have the opportunity to ask questions (4)
- I did not need to ask (5)

| Page Break |  |
| --- | --- |

Did your investigations for recurrent miscarriage take place in any of the following locations:

|  | Yes (1) | No (2) | I don't know/ I can't remember (3) |
| --- | --- | --- | --- |
| General practice (GP/Practice Nurse) (1) |  |  |  |
| Hospital-Early Pregnancy Unit (2) |  |  |  |
| Hospital-pregnancy loss clinic (3) |  |  |  |
| Hospital-recurrent miscarriage clinic (4) |  |  |  |
| Hospital-gynaecology clinic (5) |  |  |  |
| Phlebotomy clinic (6) |  |  |  |
| Private consultant rooms (7) |  |  |  |
| Fertility clinic (8) |  |  |  |
| Other. Please specify: (9) |  |  |  |

| Page Break |  |
| --- | --- |

Did you have to travel to attend these investigation(s)?

- Yes (1)
- No (2)
- Not applicable (3)
- I don't know/ I can't remember (4)

Skip To: Q38 If Did you have to travel to attend these investigation(s)? = No

Skip To: Q38 If Did you have to travel to attend these investigation(s)? = Not applicable

Skip To: Q38 If Did you have to travel to attend these investigation(s)? = I don't know/ I can't remember

| Page Break |  |
| --- | --- |

| 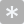 |
| --- |

How many miles or kilometres did you travel?

- Miles (21) ________________________________________________
- Kilometres (22) ________________________________________________

| Page Break |  |
| --- | --- |

How did you usually travel?

- Bus (1)
- Car (2)
- Train (3)
- Taxi (4)
- Bike (5)
- Walk (6)

| Page Break |  |
| --- | --- |

Did you spend money on travel to investigation(s)? (e.g., on petrol/diesel, public transport/ parking)

- Yes. Please specify, on average, how much you spent on travel (€) (1) ________________________________________________
- No (2)
- I don't know / I can't remember (3)
- Not applicable (4)

| Page Break |  |
| --- | --- |

Were you told that it might be helpful if someone (e.g. partner, relative) went with you to the appointment(s)?

- Yes (1)
- No (2)
- I don't know / I can't remember (3)
- Not applicable (4)

| Page Break |  |
| --- | --- |

Did anyone attend the appointments with you? (e.g., partner, family member, friend)

- Yes (1)
- No (2)
- Not facilitated due to COVID-19 restrictions (3)
- I don't know / I can't remember (4)

Skip To: Q43 If Did anyone attend the appointments with you? (e.g., partner, family member, friend) = No

Skip To: Q43 If Did anyone attend the appointments with you? (e.g., partner, family member, friend) = Not facilitated due to COVID-19 restrictions

Skip To: Q43 If Did anyone attend the appointments with you? (e.g., partner, family member, friend) = I don't know / I can't remember

| Page Break |  |
| --- | --- |

If someone attended your investigations appointment with you, what best describes their employment status at that time? 
 
*(We are asking you this question because we want to find out the impact that receiving recurrent miscarriage care has had on you and your family in many areas, including employment)*

- In paid work (1)
- Retired (2)
- In education (3)
- Not working (4)
- I don't know / I can't remember (5)
- Other. Please specify: (6) ________________________________________________

| Page Break |  |
| --- | --- |

If you have children or other dependents, did you pay someone to look after them while attending your appointment for investigation(s)?
 
*(We are asking you this question because we want to find out the impact that receiving recurrent miscarriage care has had on you and your family in many areas, including spending)*

- Yes. Please specify, on average, how much it cost for someone to look after your child(ren)/dependents per appointment (€)? (1) ________________________________________________
- No (2)
- I don't know / I can't remember (3)
- Not applicable (4)

| Page Break |  |
| --- | --- |

How would you rate the waiting area(s) where you attended for investigation?

- Very good (1)
- Good (2)
- Satisfactory (3)
- Poor (4)
- I don't know / I can't remember (5)
- Not applicable (7)

| Page Break |  |
| --- | --- |

Carry Forward All Choices – Displayed & Hidden from "How would you rate the waiting area(s) where you attended for investigation?"

| 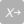 |
| --- |

How would you rate the area(s) where the investigations were carried out? (e.g., consultation room/ ultrasound scan area)

- Very good (1)
- Good (2)
- Satisfactory (3)
- Poor (4)
- I don't know / I can't remember (5)
- Not applicable (6)

| Page Break |  |
| --- | --- |

Were any of the following investigations carried out to try to find out the cause of your recurrent miscarriages?

|  | Yes (1) | No (2) | I don't know/ I can't remember (3) |
| --- | --- | --- | --- |
| Discussion of medical and family history (1) |  |  |  |
| Blood tests (this could include thyroid function tests, blood glucose (sugar) levels, antibody screen, thrombophilia screen) (2) |  |  |  |
| Ultrasound (abdominal or transvaginal) (3) |  |  |  |
| MRI (4) |  |  |  |
| Hysteroscopy (exam of the inside of the cervix and uterus using a thin, lighted, flexible tube called a hysteroscope) (5) |  |  |  |
| Genetic testing of pregnancy tissue (often called karyotyping or cytogenetics) (6) |  |  |  |
| Genetic testing of you and/or your partner (often called karyotyping) (7) |  |  |  |
| Other. Please specify: (8) |  |  |  |

| Page Break |  |
| --- | --- |

Did you pay for any of the investigations (including any appointments, or the investigations themselves) out of your own pocket? Please estimate your overall expenses.

 [Please exclude any payments covered by your private health insurer (if applicable), including reimbursements]

- Yes. Please specify, on average, how much you spent (€): (1) ________________________________________________
- No (2)
- I don't know / I can't remember (3)

| Page Break |  |
| --- | --- |

Were you told how long you would be waiting for the results of the investigations?

- Yes (1)
- No (2)
- I don't know / I can't remember (3)

| Page Break |  |
| --- | --- |

Carry Forward All Choices – Displayed & Hidden from "Were you told how long you would be waiting for the results of the investigations?"

| 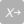 |
| --- |

Were you told who to contact if you had questions/concerns while waiting for the results of your investigations?

- Yes (1)
- No (2)
- I don't know / I can't remember (3)

| Page Break |  |
| --- | --- |

Did you have a healthcare professional that you could talk to about your worries and fears?

- Yes, always (1)
- Yes, sometimes (2)
- No (3)
- I had no worries or fears (4)
- I don't know / I can't remember (5)

| Page Break |  |
| --- | --- |

If your partner wanted to talk to a healthcare professional about the investigations, did they have enough opportunity to do so?

- Yes, definitely (1)
- Yes, to some extent (2)
- No (3)
- I did not have a partner (4)
- My partner did not want or need information (5)
- I did not want my partner to talk to a doctor (6)

| Page Break |  |
| --- | --- |

Did you have confidence and trust in those providing your care while you were being investigated?

- Yes, always (1)
- Yes, sometimes (2)
- No (3)

| Page Break |  |
| --- | --- |

Did it ever happen that one healthcare professional said one thing about investigations for recurrent miscarriage, and another said something different?

- Often (1)
- Sometimes (2)
- Only once (3)
- Never (4)
- I don't know / I can't remember (5)

| Page Break |  |
| --- | --- |

Carry Forward All Choices – Displayed & Hidden from "Did it ever happen that one healthcare professional said one thing about investigations for recurrent miscarriage, and another said something different?"

| 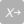 |
| --- |

Did you ever think that the healthcare professionals were deliberately not telling you certain things that you wanted to know?

- Often (1)
- Sometimes (2)
- Only once (3)
- Never (4)
- I don't know / I can't remember (5)

| Page Break |  |
| --- | --- |

Did you feel you were treated with respect and dignity while you were being investigated?

- Yes, always (1)
- Yes, sometimes (2)
- No (3)

| Page Break |  |
| --- | --- |

Do you think the healthcare professionals involved in your care did everything they could to investigate the cause of your recurrent miscarriage?

- Yes, definitely (1)
- Yes, to some extent (2)
- No (3)
- I don't know / I can't remember (4)

| Page Break |  |
| --- | --- |

**Section 3: Getting your results**
In this section, we will ask you about **receiving the results of your investigations** for recurrent miscarriage. As in the previous section, if you have been investigated multiple times, we ask that you think about **receiving the results from the first time you had investigations f**or recurrent miscarriage.

Did you receive the results of your investigations for recurrent miscarriage?

- Yes, I have received all of my results (1)
- Yes, I have received some of my results (2)
- No, I did not get any results (3)
- No, I am still waiting for all of my results (4)
- I don’t know / I can't remember (5)

Skip To: Q89 If Did you receive the results of your investigations for recurrent miscarriage? = No, I did not get any results

Skip To: Q89 If Did you receive the results of your investigations for recurrent miscarriage? = No, I am still waiting for all of my results

Skip To: Q89 If Did you receive the results of your investigations for recurrent miscarriage? = I don’t know / I can't remember

| Page Break |  |
| --- | --- |

Carry Forward All Choices – Displayed & Hidden from "After referral, how long did you wait for an appointment for investigation(s) for your recurrent miscarriage?"

| 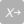 |
| --- |

How long did you wait to get the results of your investigations for recurrent miscarriage?

- Less than one month (1)
- 1-2 months (2)
- 3-4 months (3)
- 5-6 months (4)
- 7-12 months (5)
- More than 12 months (6)
- I don't know / I can't remember (7)

| Page Break |  |
| --- | --- |

How did you feel about the length of time you waited for the results?

- Too short (1)
- About right (2)
- Too long (3)
- I don't know / I can't remember (4)

| Page Break |  |
| --- | --- |

Did you receive the results of the investigations from any of the following locations:

|  | Yes (1) | No (2) | I don't know/ I can't remember (3) |
| --- | --- | --- | --- |
| General practice (GP/Practice Nurse) (1) |  |  |  |
| Hospital-pregnancy loss clinic (2) |  |  |  |
| Hospital-recurrent miscarriage clinic (3) |  |  |  |
| Hospital-gynaecology clinic (4) |  |  |  |
| Private consultant rooms (5) |  |  |  |
| Fertility clinic (6) |  |  |  |
| Other. Please specify: (7) |  |  |  |

| Page Break |  |
| --- | --- |

Did you receive the results of the investigations from any of the following health professionals?

|  | Yes (1) | No (2) | I don't know/ I can't remember (3) |
| --- | --- | --- | --- |
| GP (1) |  |  |  |
| Midwife/ nurse in the hospital (2) |  |  |  |
| Sonographer (healthcare professional who specialises in the use of ultrasound) (3) |  |  |  |
| Consultant in a public hospital (4) |  |  |  |
| Doctor in a public hospital (5) |  |  |  |
| Private consultant (6) |  |  |  |
| Doctor/midwife/nurse at a fertility clinic (7) |  |  |  |
| Administrator / Secretary (8) |  |  |  |
| Other, Please specify: (9) |  |  |  |

| Page Break |  |
| --- | --- |

How were the results of your investigations given to you?

|  | Yes (1) | No (2) | Don't know (3) |
| --- | --- | --- | --- |
| Phone (1) |  |  |  |
| Email (2) |  |  |  |
| Letter (3) |  |  |  |
| Face to Face appointment (4) |  |  |  |
| Virtual appointment (videoconferencing) (5) |  |  |  |
| Other. Please specify: (6) |  |  |  |

| Page Break |  |
| --- | --- |

Did you take time from work to receive the results of your investigations?

- Yes. Please specify, on average, how many hours you took off from work (1) ________________________________________________
- No (2)
- Not applicable (3)
- I don't know / I can't remember (4)

Skip To: Q66 If Did you take time from work to receive the results of your investigations? = No

Skip To: Q66 If Did you take time from work to receive the results of your investigations? = I don't know / I can't remember

Skip To: Q66 If Did you take time from work to receive the results of your investigations? = Not applicable

| Page Break |  |
| --- | --- |

Carry Forward All Choices – Displayed & Hidden from "Was your job/work performance affected by attending your appointment for investigations? "

| 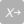 |
| --- |

Was your job/work performance affected by attending your appointment to receive the results of your investigations?

- Missed a chance of promotion (1)
- Missed a deadline (2)
- No, it was not affected (3)
- I don’t know / I can't remember (4)
- Other. Please specify: (5) ________________________________________________

| Page Break |  |
| --- | --- |

If you attended a face-to-face appointment, did you have to travel to receive the results of your investigations? (e.g., distance from home to your appointment)

- Yes (1)
- No (2)
- Not applicable (3)
- I don't know/ I can't remember (4)

Skip To: Q67 If If you attended a face-to-face appointment, did you have to travel to receive the results of your... = No

Skip To: Q67 If If you attended a face-to-face appointment, did you have to travel to receive the results of your... = Not applicable

Skip To: Q67 If If you attended a face-to-face appointment, did you have to travel to receive the results of your... = I don't know/ I can't remember

| Page Break |  |
| --- | --- |

| 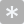 |
| --- |

How many miles or kilometres did you travel?

- Miles (4) ________________________________________________
- Kilometres (6) ________________________________________________

| Page Break |  |
| --- | --- |

Carry Forward All Choices – Displayed & Hidden from "How did you usually travel? "

| 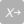 |
| --- |

How did you usually travel?

- Bus (1)
- Car (2)
- Train (3)
- Taxi (4)
- Bike (5)
- Walk (6)

| Page Break |  |
| --- | --- |

Carry Forward All Choices – Displayed & Hidden from "Did you spend money on travel to investigation(s)? (e.g., on petrol/diesel, public transport/ parking)"

| 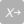 |
| --- |

Did you spend money on travel to receive the results of your investigations? (e.g., petrol/diesel, public transport/ parking)

- Yes. Please specify, on average, how much you spent on travel (€) (1) ________________________________________________
- No (2)
- I don't know / I can't remember (3)
- Not applicable (4)

| Page Break |  |
| --- | --- |

Carry Forward All Choices – Displayed & Hidden from "Were you told that it might be helpful if someone (e.g. partner, relative) went with you to the appointment(s)?"

| 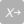 |
| --- |

Were you told that it might be helpful to have someone (e.g. partner, relative) with you when you received your test results?

- Yes (1)
- No (2)
- I don't know / I can't remember (3)
- Not applicable (4)

| Page Break |  |
| --- | --- |

Carry Forward All Choices – Displayed & Hidden from "Did anyone attend the appointments with you? (e.g., partner, family member, friend)"

| 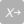 |
| --- |

Did anyone attend with you to receive your investigation results? (e.g., partner, family member, friend)

- Yes (1)
- No (2)
- Not facilitated due to COVID-19 restrictions (3)
- I don't know / I can't remember (4)

Skip To: Q72 If Condition: No Is Selected. Skip to: If you have children or other depende....

Skip To: Q72 If Condition: Not facilitated due to COVI... Is Selected. Skip to: If you have children or other depende....

Skip To: Q72 If Condition: I don't know / I can't reme... Is Selected. Skip to: If you have children or other depende....

| Page Break |  |
| --- | --- |

Carry Forward All Choices – Displayed & Hidden from "If someone attended your investigations appointment with you, what best describes their employment status at that time?   (We are asking you this question because we want to find out the impact that receiving recurrent miscarriage care has had on you and your family in many areas, including employment)"

| 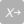 |
| --- |

If someone attended to receive your investigation results with you, what best describes their employment status at that time?
 
*(We are asking you this question because we want to find out the impact that receiving recurrent miscarriage care has had on you and your family in many areas, including employment)*

- In paid work (1)
- Retired (2)
- In education (3)
- Not working (4)
- I don't know / I can't remember (5)
- Other. Please specify: (6) ________________________________________________

| Page Break |  |
| --- | --- |

Carry Forward All Choices – Displayed & Hidden from "If you have children or other dependents, did you pay someone to look after them while attending your appointment for investigation(s)?   (We are asking you this question because we want to find out the impact that receiving recurrent miscarriage care has had on you and your family in many areas, including spending)"

| 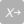 |
| --- |

If you have children or other dependents, did you pay someone to look after them while receiving your investigation results?
 
*(We are asking you this question because we want to find out the impact that receiving recurrent miscarriage care has had on you and your family in many areas, including spending)*

- Yes. Please specify, on average, how much it cost for someone to look after your child(ren)/dependents per appointment (€)? (1) ________________________________________________
- No (2)
- I don't know / I can't remember (3)
- Not applicable (4)

| Page Break |  |
| --- | --- |

Carry Forward All Choices – Displayed & Hidden from "Did you pay for any of the investigations (including any appointments, or the investigations themselves) out of your own pocket? Please estimate your overall expenses. [Please exclude any payments covered by your private health insurer (if applicable), including reimbursements]"

| 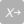 |
| --- |

Did you have to pay for any of the visits (to receive your investigation results) out of your own pocket? Please estimate your overall expenses.

 [Please exclude any payments covered by your private health insurer (if applicable), including reimbursements]

- Yes. Please specify, on average, how much you spent (€): (1) ________________________________________________
- No (2)
- I don't know / I can't remember (3)

| Page Break |  |
| --- | --- |

Carry Forward All Choices – Displayed & Hidden from "How would you rate the waiting area(s) where you attended for investigation?"

| 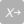 |
| --- |

How would you rate the waiting area(s) where you attended to receive your results?

- Very good (1)
- Good (2)
- Satisfactory (3)
- Poor (4)
- I don't know / I can't remember (5)
- Not applicable (6)

| Page Break |  |
| --- | --- |

Carry Forward All Choices – Displayed & Hidden from "How would you rate the area(s) where the investigations were carried out? (e.g., consultation room/ ultrasound scan area)"

| 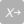 |
| --- |

How would you rate the area(s) where you were given your results? (e.g., consultation room/ ultrasound scan area)

- Very good (1)
- Good (2)
- Satisfactory (3)
- Poor (4)
- I don't know / I can't remember (5)
- Not applicable (6)

| Page Break |  |
| --- | --- |

Were the results explained to you in a way that you could understand?

- Yes, completely (1)
- Yes, to some extent (2)
- No (3)
- I don't know / I can't remember (4)
- Not applicable (5)

| Page Break |  |
| --- | --- |

Carry Forward All Choices – Displayed & Hidden from "Did you receive enough information about investigations for recurrent miscarriage?"

| 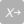 |
| --- |

Did you receive enough information about the results?

- Yes, definitely (1)
- Yes, to some extent (2)
- No (3)
- I did not want or need this information (4)
- I don't know / I can't remember (5)

| Page Break |  |
| --- | --- |

Carry Forward All Choices – Displayed & Hidden from "Were you given any written information about investigations for recurrent miscarriage?"

| 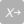 |
| --- |

Were you given any written information about the results?

- Yes (1)
- No (2)
- I did not want or need any written or printed information (3)
- I don’t know / I can’t remember (4)

| Page Break |  |
| --- | --- |

Carry Forward All Choices – Displayed & Hidden from "Did you feel you had enough time to discuss investigations for recurrent miscarriage, and what they would involve?"

| 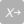 |
| --- |

Did you feel you had enough time to discuss the results?

- Yes, definitely (1)
- Yes, to some extent (2)
- No (3)
- I don't know / I can't remember (4)

| Page Break |  |
| --- | --- |

Carry Forward All Choices – Displayed & Hidden from "Were you told who to contact if you had questions/concerns while waiting for the results of your investigations?"

| 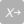 |
| --- |

Were you told who to contact if you had questions about your results?

- Yes (1)
- No (2)
- I don't know / I can't remember (3)

| Page Break |  |
| --- | --- |

Carry Forward All Choices – Displayed & Hidden from "When you had questions to ask about investigations for recurrent miscarriage, did you get answers that you could understand?"

| 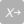 |
| --- |

When you had questions to ask about your results, did you get answers that you could understand?

- Yes, always (1)
- Yes, sometimes (2)
- No (3)
- I did not have the opportunity to ask questions (4)
- I did not need to ask (5)

| Page Break |  |
| --- | --- |

Carry Forward All Choices – Displayed & Hidden from "Did you have a healthcare professional that you could talk to about your worries and fears?"

| 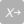 |
| --- |

Did you have a healthcare professional that you could talk to about your worries and fears?

- Yes, always (1)
- Yes, sometimes (2)
- No (3)
- I had no worries or fears (4)
- I don't know / I can't remember (5)

| Page Break |  |
| --- | --- |

Carry Forward All Choices – Displayed & Hidden from "If your partner wanted to talk to a healthcare professional about the investigations, did they have enough opportunity to do so?"

| 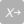 |
| --- |

If your partner wanted to talk to a healthcare professional, about the results did they have enough opportunity to do so?

- Yes, definitely (1)
- Yes, to some extent (2)
- No (3)
- I did not have a partner (4)
- My partner did not want or need information (5)
- I did not want my partner to talk to a doctor (6)

| Page Break |  |
| --- | --- |

Did it ever happen that one healthcare professional said one thing about your results and another said something different?

- Often (1)
- Sometimes (2)
- Only once (3)
- Never (4)
- I don't know/ I can't remember (5)

| Page Break |  |
| --- | --- |

Carry Forward All Choices – Displayed & Hidden from "Did you ever think that the healthcare professionals were deliberately not telling you certain things that you wanted to know?"

| 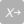 |
| --- |

Did you ever think that healthcare professionals were deliberately not telling you certain things that you wanted to know about your results?

- Often (1)
- Sometimes (2)
- Only once (3)
- Never (4)
- I don't know / I can't remember (5)

| Page Break |  |
| --- | --- |

Did you have confidence and trust in those providing your care?

- Yes, always (1)
- Yes, sometimes (2)
- No (3)

| Page Break |  |
| --- | --- |

Carry Forward All Choices – Displayed & Hidden from "Did you have confidence and trust in those providing your care?"

| 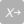 |
| --- |

Did you feel you were treated with respect and dignity?

- Yes, always (1)
- Yes, sometimes (2)
- No (3)

| Page Break |  |
| --- | --- |

Did the results provide a possible explanation(s)/reason(s) for your recurrent miscarriages?

- Yes (1)
- No (2)
- I don't know / I don't remember (3)

| Page Break |  |
| --- | --- |

**Section 4: Treatment/Plan of Care**
In this section, we will ask you about **any plans put in place for a future pregnancy** following initial investigations for recurrent miscarriage

Was a treatment plan/plan of care put in place for future pregnancy?

- Yes (1)
- No (2)
- I don’t know/can’t remember (3)
- Not applicable (4)

Skip To: Q108 If Was a treatment plan/plan of care put in place for future pregnancy? = No

Skip To: Q108 If Was a treatment plan/plan of care put in place for future pregnancy? = I don’t know/can’t remember

Skip To: Q108 If Was a treatment plan/plan of care put in place for future pregnancy? = Not applicable

| Page Break |  |
| --- | --- |

Did you receive your treatment plan/plan of care from any of the following locations?

|  | Yes (5) | No (6) | I don't know/ I cam't remember (7) |
| --- | --- | --- | --- |
| GP (1) |  |  |  |
| Hospital-general (2) |  |  |  |
| Hospital-pregnancy loss clinic (3) |  |  |  |
| Hospital-recurrent miscarriage clinic (4) |  |  |  |
| Hospital-gynaecology clinic (5) |  |  |  |
| Private Consultant Rooms (6) |  |  |  |
| Fertility clinic (7) |  |  |  |
| Other, please specify (8) |  |  |  |

| Page Break |  |
| --- | --- |

Did any of the following healthcare professionals provide you with a plan of care for a future pregnancy?

|  | Yes (1) | No (2) | I don't know/ I can't remember (3) |
| --- | --- | --- | --- |
| GP (1) |  |  |  |
| Midwife/nurse in the hospital (2) |  |  |  |
| Sonographer (healthcare professional who specialises in the use of ultrasound) (3) |  |  |  |
| Consultant in a public hospital (4) |  |  |  |
| Doctor in a public hospital (5) |  |  |  |
| Private consultant (6) |  |  |  |
| Doctor/midwife/nurse at a fertility clinic (7) |  |  |  |
| Other, please specify (8) |  |  |  |

| Page Break |  |
| --- | --- |

Were the risks and benefits of treatment/plan of care explained in a way you could understand?

- Yes, completely (1)
- Yes, to some extent (2)
- No (3)

| Page Break |  |
| --- | --- |

Were you involved as much as you wanted to be in decisions about your treatment/plan of care?

- Yes, definitely (1)
- Yes, to some extent (2)
- No (3)

| Page Break |  |
| --- | --- |

Did you receive enough information about the treatment plan/plan of care?

- Yes, definitely (1)
- Yes, to some extent (2)
- No (3)
- I did not want or need this information (4)
- I don’t know / I can’t remember (5)

| Page Break |  |
| --- | --- |

Were you given any written information about the treatment plan/plan of care?

- Yes (1)
- No (2)
- I did not want or need any written or printed information (3)
- I don’t know / I can’t remember (4)

| Page Break |  |
| --- | --- |

Did you feel you had enough time to discuss the treatment plan/plan of care?

- Yes, definitely (1)
- Yes, to some extent (2)
- No (3)

| Page Break |  |
| --- | --- |

Were you told who to contact if you had questions about your treatment plan/plan of care?

- Yes (1)
- No (2)
- I don’t know / can’t remember (3)

| Page Break |  |
| --- | --- |

When you had questions to ask about your treatment plan/plan of care, did you get answers that you could understand?

- Yes, always (1)
- Yes, sometimes, (2)
- No (3)
- I did not have the opportunity to ask questions (4)
- I did not need to ask (5)

| Page Break |  |
| --- | --- |

Did you have a healthcare professional that you could talk to about your worries and fears?

- Yes, always (1)
- Yes, sometimes (2)
- No (3)
- I had no worries or fears (4)
- I don’t know / can’t remember (5)

| Page Break |  |
| --- | --- |

If your partner wanted to talk to a healthcare professional, about the treatment plan/plan of care did they have enough opportunity to do so?

- Yes, definitely (1)
- Yes, to some extent (2)
- No (3)
- No partner was involved (4)
- I did not have a partner (5)
- My partner did not want or need information (6)
- I did not want my partner to talk to a doctor (7)

| Page Break |  |
| --- | --- |

Did it ever happen that one healthcare professional said one thing about your treatment plan/plan of care and another said something different?

- Often (1)
- Sometimes (2)
- Only once (3)
- Never (4)
- I don’t know / can’t remember (5)

| Page Break |  |
| --- | --- |

Carry Forward All Choices – Displayed & Hidden from "Did it ever happen that one healthcare professional said one thing about your treatment plan/plan of care and another said something different?"

| 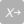 |
| --- |

Did you ever think that healthcare professionals were deliberately not telling you certain things that you wanted to know about your treatment plan/plan of care?

- Often (1)
- Sometimes (2)
- Only once (3)
- Never (4)
- I don’t know / can’t remember (5)

| Page Break |  |
| --- | --- |

Did you have confidence and trust in those providing your care?

- Yes, always (1)
- Yes, sometimes (2)
- No (3)

| Page Break |  |
| --- | --- |

Did you feel you were treated with respect and dignity?

- Yes, always (1)
- Yes, sometimes (2)
- No (3)

| Page Break |  |
| --- | --- |

Do you think the healthcare professionals involved did everything they could to treat your recurrent miscarriages?

- Yes, definitely (1)
- Yes, to some extent (2)
- No (3)
- I don’t know / I can’t remember (4)

| Page Break |  |
| --- | --- |

**Section 5: Care in Subsequent Pregnancy**
In this section, we will ask you about the **care you received if you got pregnant again** following initial investigations for recurrent miscarriage. We are particularly interested in the **pregnancy care you received because of your history of recurrent miscarriage**.

Did you experience another pregnancy?

- Yes (1)
- No (2)

Skip To: Q274 If Did you experience another pregnancy? = No

| Page Break |  |
| --- | --- |

Were you offered early/reassurance ultrasound scans in a subsequent pregnancy?

- Yes (1)
- No (2)
- I don't know / I can't remember (3)

Skip To: Q113 If Were you offered early/reassurance ultrasound scans in a subsequent pregnancy? = No

Skip To: Q113 If Were you offered early/reassurance ultrasound scans in a subsequent pregnancy? = I don't know / I can't remember

| Page Break |  |
| --- | --- |

How many early reassurance scans did you have? [Please select from drop-down list]

▼ 0 (1) ... I don't know/ I can't remember (12)

| Page Break |  |
| --- | --- |

Did you have to pay for any of the early reassurance scans out of your own pocket? Please estimate your overall expenses.

 [Please exclude any payments covered by your private health insurer (if applicable), including reimbursements]

- Yes. Please specify, on average, how much you spent on early reassurance scans (€) (1) ________________________________________________
- No (2)
- I don't know / I can't remember (3)

| Page Break |  |
| --- | --- |

Thinking particularly about the care you received because of your recurrent miscarriages, in your subsequent pregnancy did you receive care in any of the following locations?

|  | Yes (1) | No (2) | I don't know/ I can't remember (3) |
| --- | --- | --- | --- |
| General practice (GP/Practice Nurse) (1) |  |  |  |
| Hospital-general (2) |  |  |  |
| Hospital-pregnancy loss clinic (3) |  |  |  |
| Hospital-recurrent miscarriage clinic (4) |  |  |  |
| Hospital-early pregnancy assessment unit (9) |  |  |  |
| Hospital-gynaecology clinic (5) |  |  |  |
| Private Consultant Rooms (6) |  |  |  |
| Fertility clinic (7) |  |  |  |
| Other. Please specify: (8) |  |  |  |

| Page Break |  |
| --- | --- |

| Page Break |  |
| --- | --- |

Did you have to travel to receive early reassurance scan (e.g., distance from home to your appointment)

- Yes (1)
- No (2)
- Not applicable (3)
- I don't know/ I can't remember (4)

Skip To: Q115 If Did you have to travel to receive early reassurance scan (e.g., distance from home to your appoin... = No

Skip To: Q115 If Did you have to travel to receive early reassurance scan (e.g., distance from home to your appoin... = Not applicable

Skip To: Q115 If Did you have to travel to receive early reassurance scan (e.g., distance from home to your appoin... = I don't know/ I can't remember

| Page Break |  |
| --- | --- |

| 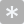 |
| --- |

How many miles or kilometres did you travel?

- Miles (4) ________________________________________________
- Kilometres (5) ________________________________________________

| Page Break |  |
| --- | --- |

Carry Forward All Choices – Displayed & Hidden from "How did you usually travel? "

| 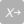 |
| --- |

How did you usually travel?

- Bus (1)
- Car (2)
- Train (3)
- Taxi (4)
- Bike (5)
- Walk (6)

| Page Break |  |
| --- | --- |

Carry Forward All Choices – Displayed & Hidden from "Did you spend money on travel to receive the results of your investigations? (e.g., petrol/diesel, public transport/ parking)"

| 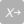 |
| --- |

Did you spend money on travel to receive subsequent pregnancy care? (e.g., public transport/ parking)

- Yes. Please specify, on average, how much you spent on travel (€) (1) ________________________________________________
- No (2)
- I don't know / I can't remember (3)
- Not applicable (4)

| Page Break |  |
| --- | --- |

Carry Forward All Choices – Displayed & Hidden from "Did you take time off from work to attend your appointment for investigations?"

| 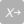 |
| --- |

Did you take time from work to receive early/reassurance scans in a subsequent pregnancy?

- Yes. Please specify, on average, how many hours you took off from work (1) ________________________________________________
- No (2)
- I don't know / I can't remember (3)
- Not applicable (4)

Skip To: Q119 If Condition: No Is Selected. Skip to: Were you told who to contact if you h....

Skip To: Q119 If Condition: I don't know / I can't reme... Is Selected. Skip to: Were you told who to contact if you h....

Skip To: Q119 If Condition: Not applicable Is Selected. Skip to: Were you told who to contact if you h....

| Page Break |  |
| --- | --- |

Carry Forward All Choices – Displayed & Hidden from "Was your job/work performance affected by attending your appointment for investigations? "

| 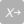 |
| --- |

Was your job/work performance affected by attending early/reassurance scans/ receiving subsequent pregnancy care? [Pick the most relevant]

- Missed a chance of promotion (1)
- Missed a deadline (2)
- No, it was not affected (3)
- I don’t know / I can't remember (4)
- Other. Please specify: (5) ________________________________________________

| Page Break |  |
| --- | --- |

Were you told who to contact if you had questions about care in your subsequent pregnancy?

- Yes (1)
- No (2)
- I don't know/ I can't remember (3)

| Page Break |  |
| --- | --- |

Carry Forward All Choices – Displayed & Hidden from "If your partner wanted to talk to a healthcare professional, about the results did they have enough opportunity to do so?"

| 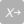 |
| --- |

If your partner wanted to talk to a healthcare professional, did they have enough opportunity to do so?

- Yes, definitely (1)
- Yes, to some extent (2)
- No (3)
- I did not have a partner (4)
- My partner did not want or need information (5)
- I did not want my partner to talk to a doctor (6)

| Page Break |  |
| --- | --- |

Were you told that it might be helpful if someone (e.g. partner, relative) went with you to your ultrasound scans and/or hospital appointment(s)?

- Yes (1)
- No (2)
- I don't know/ I can't remember (3)

| Page Break |  |
| --- | --- |

Carry Forward All Choices – Displayed & Hidden from "Did anyone attend with you to receive your investigation results? (e.g., partner, family member, friend)"

| 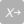 |
| --- |

Did anyone attend with you to receive extra scans or hospital visits? (e.g., partner, family member, friend)

- Yes (1)
- No (2)
- Not facilitated due to COVID-19 restrictions (3)
- I don't know / I can't remember (4)

Skip To: Q124 If Condition: No Is Selected. Skip to: If you have children or other depende....

Skip To: Q124 If Condition: Not facilitated due to COVI... Is Selected. Skip to: If you have children or other depende....

Skip To: Q124 If Condition: I don't know / I can't reme... Is Selected. Skip to: If you have children or other depende....

| Page Break |  |
| --- | --- |

Carry Forward All Choices – Displayed & Hidden from "If someone attended your investigations appointment with you, what best describes their employment status at that time?   (We are asking you this question because we want to find out the impact that receiving recurrent miscarriage care has had on you and your family in many areas, including employment)"

| 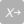 |
| --- |

If someone attended extra scans or hospital visits with you, what best describes their employment status? 
 
*(We are asking you this question because we want to find out the impact that receiving recurrent miscarriage care has had on you and your family in many areas, including employment)*

- In paid work (1)
- Retired (2)
- In education (3)
- Not working (4)
- I don't know / I can't remember (5)
- Other. Please specify: (6) ________________________________________________

| Page Break |  |
| --- | --- |

Carry Forward All Choices – Displayed & Hidden from "If you have children or other dependents, did you pay someone to look after them while attending your appointment for investigation(s)?   (We are asking you this question because we want to find out the impact that receiving recurrent miscarriage care has had on you and your family in many areas, including spending)"

| 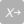 |
| --- |

If you have children or other dependents, did you pay someone to look after them while receiving your ultrasound scans and/or hospital appointment(s)?
 
*(We are asking you this question because we want to find out the impact that receiving recurrent miscarriage care has had on you and your family in many areas, including spending)*

- Yes. Please specify, on average, how much it cost for someone to look after your child(ren)/dependents per appointment (€)? (1) ________________________________________________
- No (2)
- I don't know / I can't remember (3)
- Not applicable (4)

| Page Break |  |
| --- | --- |

Carry Forward All Choices – Displayed & Hidden from "Did you feel you were treated with respect and dignity?"

| 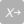 |
| --- |

Did you feel you were treated with respect and dignity?

- Yes, always (1)
- Yes, sometimes (2)
- No (3)

| Page Break |  |
| --- | --- |

Carry Forward All Choices – Displayed & Hidden from "How would you rate the waiting area(s) where you attended for investigation?"

| 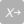 |
| --- |

How would you rate the waiting area(s) where you attended for subsequent pregnancy care?

- Very good (1)
- Good (2)
- Satisfactory (3)
- Poor (4)
- I don't know / I can't remember (5)
- Not applicable (6)

| Page Break |  |
| --- | --- |

Carry Forward All Choices – Displayed & Hidden from "How would you rate the area(s) where the investigations were carried out? (e.g., consultation room/ ultrasound scan area)"

| 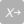 |
| --- |

How would you rate the area(s) where you attended for subsequent pregnancy care? (e.g., consultation room/ ultrasound scan area)

- Very good (1)
- Good (2)
- Satisfactory (3)
- Poor (4)
- I don't know / I can't remember (5)
- Not applicable (6)

| Page Break |  |
| --- | --- |

Were you involved as much as you wanted to be in decisions about your subsequent pregnancy care?

- Yes, definitely (1)
- Yes, to some extent (2)
- No (3)

| Page Break |  |
| --- | --- |

Carry Forward All Choices – Displayed & Hidden from "Did you feel you had enough time to discuss investigations for recurrent miscarriage, and what they would involve?"

| 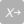 |
| --- |

Did you feel you had enough time to discuss your care in your subsequent pregnancy?

- Yes, definitely (1)
- Yes, to some extent (2)
- No (3)
- I don't know / I can't remember (4)

| Page Break |  |
| --- | --- |

| Page Break |  |
| --- | --- |

Carry Forward All Choices – Displayed & Hidden from "When you had questions to ask about your results, did you get answers that you could understand?"

| 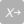 |
| --- |

When you had questions to ask about a subsequent pregnancy and recurrent miscarriage, did you get answers that you could understand?

- Yes, always (1)
- Yes, sometimes (2)
- No (3)
- I did not have the opportunity to ask questions (4)
- I did not need to ask (5)

| Page Break |  |
| --- | --- |

Carry Forward All Choices – Displayed & Hidden from "Did you have a healthcare professional that you could talk to about your worries and fears?"

| 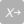 |
| --- |

Did you have a healthcare professional that you could talk to about your worries and fears?

- Yes, always (1)
- Yes, sometimes (2)
- No (3)
- I had no worries or fears (4)
- I don't know / I can't remember (5)

| Page Break |  |
| --- | --- |

Carry Forward All Choices – Displayed & Hidden from "Did you have confidence and trust in those providing your care?"

| 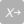 |
| --- |

Did you have confidence and trust in those providing your care?

- Yes, always (1)
- Yes, sometimes (2)
- No (3)

| Page Break |  |
| --- | --- |

Carry Forward All Choices – Displayed & Hidden from "Did it ever happen that one healthcare professional said one thing about investigations for recurrent miscarriage, and another said something different?"

| 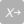 |
| --- |

Did it ever happen that one healthcare professional said one thing about your care in your subsequent pregnancy and another said something different?

- Often (1)
- Sometimes (2)
- Only once (3)
- Never (4)
- I don't know / I can't remember (5)

| Page Break |  |
| --- | --- |

Carry Forward All Choices – Displayed & Hidden from "Did you ever think that the healthcare professionals were deliberately not telling you certain things that you wanted to know?"

| 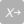 |
| --- |

Did you ever think that healthcare professionals were deliberately not telling you certain things that you wanted to know about care in your subsequent pregnancy?

- Often (1)
- Sometimes (2)
- Only once (3)
- Never (4)
- I don't know / I can't remember (5)

| Page Break |  |
| --- | --- |

Do you think the healthcare professionals involved did everything they could to support you in your subsequent pregnancy?

- Yes, definitely (1)
- Yes, to some extent (2)
- No (3)
- Definitely not (4)
- I don't know / I can't remember (5)

| Page Break |  |
| --- | --- |

| Page Break |  |
| --- | --- |

**Section 6: Information and Support Services**
In this section, we will ask you about your **experience of information and support services** for recurrent miscarriage. As in the previous section, if you have been investigated/ treated multiple times, we ask that you think about**your experience of information and support services the first time you had investigations/ treatment** for recurrent miscarriage.

Were you told about/given information about any of the following supports for recurrent miscarriage?

|  | Yes (1) | No (2) | I don't know/ I can't remember (3) |
| --- | --- | --- | --- |
| Clinical Midwife/Nurse Specialist in Bereavement and Loss (1) |  |  |  |
| Miscarriage Association of Ireland (2) |  |  |  |
| Féileacáin (Stillbirth and Neonatal Death Association of Ireland) (3) |  |  |  |
| Online discussion boards/forums (4) |  |  |  |
| Chaplaincy and/or pastoral care (5) |  |  |  |
| Religious or spiritual support group (6) |  |  |  |
| One-on-one counselling (i.e. - with a Therapist, Counsellor, Psychologist) (7) |  |  |  |
| Psychiatry (8) |  |  |  |
| Social work service (9) |  |  |  |
| Perinatal mental health (10) |  |  |  |
| Books/booklets/leaflets/pamphlets (11) |  |  |  |
| Pregnancy and Infant Loss website www.pregnancyandinfantloss.ie (12) |  |  |  |
| Cork Miscarriage website www.corkmiscarriage.com (13) |  |  |  |
| Other. Please specify: (14) |  |  |  |

| Page Break |  |
| --- | --- |

If you used any of the following supports, please indicate how helpful they have been. [Please select 'did not use' if you did not access any of the following]

|  | Extremely helpful (1) | Very helpful (2) | Somewhat helpful (3) | Not very helpful (4) | Not at all helpful (5) | Not sure (6) | Did not use (7) |
| --- | --- | --- | --- | --- | --- | --- | --- |
| Clinical Midwife/Nurse Specialist in Bereavement and Loss (1) |  |  |  |  |  |  |  |
| Miscarriage Association of Ireland (2) |  |  |  |  |  |  |  |
| Féileacáin (Stillbirth and Neonatal Death Association of Ireland) (3) |  |  |  |  |  |  |  |
| Online discussion boards/forums (4) |  |  |  |  |  |  |  |
| Chaplaincy and/or pastoral care (5) |  |  |  |  |  |  |  |
| Religious or spiritual support group (6) |  |  |  |  |  |  |  |
| One-on-one counselling (i.e. - with a Therapist, Counsellor, Psychologist) (7) |  |  |  |  |  |  |  |
| Psychiatry (8) |  |  |  |  |  |  |  |
| Social work service (9) |  |  |  |  |  |  |  |
| Perinatal mental health (10) |  |  |  |  |  |  |  |
| Books/booklets/leaflets/pamphlets (11) |  |  |  |  |  |  |  |
| Pregnancy and Infant Loss website www.pregnancyandinfantloss.ie (12) |  |  |  |  |  |  |  |
| Cork Miscarriage website www.corkmiscarriage.com (13) |  |  |  |  |  |  |  |
| Other (14) |  |  |  |  |  |  |  |

| Page Break |  |
| --- | --- |

Did you have to pay for any support services out of your own pocket? Please estimate your overall expenses. 


Please exclude any payments covered by your private health insurer (if applicable), including reimbursements.

- Yes. Please specify, on average how much you sent on support service? (€) (1) ________________________________________________
- No (2)
- I don't know / I can't remember (3)

| Page Break |  |
| --- | --- |

Did you take time from work to engage with support services? (e.g., appointment with bereavement midwife, support group meeting)

- Yes. Please specify, on average, how many hours you took off from work (1) ________________________________________________
- No (2)
- Not applicable (3)
- I don’t know / I can’t remember (4)

Skip To: Q146 If Did you take time from work to engage with support services? (e.g., appointment with bereavement... = Not applicable

Skip To: Q146 If Did you take time from work to engage with support services? (e.g., appointment with bereavement... = No

Skip To: Q146 If Did you take time from work to engage with support services? (e.g., appointment with bereavement... = I don’t know / I can’t remember

| Page Break |  |
| --- | --- |

Was your job/work performance affected by attending support services?

- Missed a chance of promotion (1)
- Missed a deadline (2)
- No, it was not affected (3)
- I don’t know / I can’t remember (4)
- Other. Please specify (5) ________________________________________________

| Page Break |  |
| --- | --- |

Carry Forward All Choices – Displayed & Hidden from "Did you have to travel to attend these investigation(s)? "

| 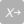 |
| --- |

Did you travel to attend support services? (e.g., distance from home to your appointment)

- Yes (1)
- No (2)
- Not applicable (3)
- I don't know/ I can't remember (4)

Skip To: Q147 If Condition: No Is Selected. Skip to: How did you usually travel? Tick one ....

Skip To: Q147 If Condition: Not applicable Is Selected. Skip to: How did you usually travel? Tick one ....

Skip To: Q147 If Condition: I don't know/ I can't remember Is Selected. Skip to: How did you usually travel? Tick one ....

| Page Break |  |
| --- | --- |

| 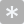 |
| --- |

How many miles or kilometres did you travel?

- Miles (4) ________________________________________________
- Kilometres (5) ________________________________________________

| Page Break |  |
| --- | --- |

How did you usually travel?

- Bus (1)
- Car (2)
- Train (3)
- Taxi (4)
- Bike (5)
- Walk (6)
- I did not travel (7)

| Page Break |  |
| --- | --- |

Did you spend on travel to attend support services? (e.g., petrol/diesel, public transport/ parking)

- Yes. Please specify, on average, how much you spent on travel (€) to support services? (1) ________________________________________________
- No (2)
- Not applicable (3)
- I don’t know/ I can’t remember (4)

| Page Break |  |
| --- | --- |

Did anyone access support services with you? (e.g., partner, family member, friend)

- Yes (1)
- No (2)
- Not facilitated due to COVID-19 restrictions (3)
- I don’t know/can’t remember (4)
- Not applicable, I did not access support services (6)

Skip To: Q151 If Did anyone access support services with you? (e.g., partner, family member, friend) = No

Skip To: Q151 If Did anyone access support services with you? (e.g., partner, family member, friend) = Not facilitated due to COVID-19 restrictions

Skip To: Q151 If Did anyone access support services with you? (e.g., partner, family member, friend) = I don’t know/can’t remember

Skip To: Q137 If Did anyone access support services with you? (e.g., partner, family member, friend) = Not applicable, I did not access support services

| Page Break |  |
| --- | --- |

If someone attended support services with you, what best describes their employment status? 
 
(W*e are asking you this question because we want to find out the impact that receiving recurrent miscarriage care has had on you and your family in many areas, including employment)*

- In paid work (1)
- Retired (2)
- In education (3)
- Not working (4)
- Other. Please specify (5) ________________________________________________
- I don’t know/can’t remember (6)

| Page Break |  |
| --- | --- |

If you have children or other dependents, did you pay someone to look after them while attending support services?
 
*(We are asking you this question because we want to find out the impact that receiving recurrent miscarriage care has had on you and your family in many areas, including spending)*

- Yes. Please specify, on average. how much did you spent (€) (1) ________________________________________________
- No (2)
- Not applicable (3)
- I don’t know/can’t remember (4)

| Page Break |  |
| --- | --- |

**Section 7: Use of other health services**
 In this section, we will ask you to **estimate other healthcare service** use such as a visit to your GP during your recurrent miscarriage care. If you have been **investigated/ treated multiple times,** we ask that you think about **the other health services you used during the time when you first had investigations/treatment.**
 

Did you attend primary care appointments? (e.g., primary care appointments include a visit to a GP and/or practice nurse in your GP practice)

- Yes. Please estimate, how many primary care appointments you attended (1) ________________________________________________
- No (2)
- Not applicable (3)
- I don't know/ I can't remember (4)

| Page Break |  |
| --- | --- |

Did you attend other appointments? (e.g., alternative therapies, private scans that you sought out on your own behalf, in addition to scans provided by the hospital/clinic)

- Yes (1)
- No (2)
- Not applicable (3)
- I don't know/ I can't remember (4)

Skip To: Q152 If Did you attend other appointments? (e.g., alternative therapies, private scans that you sought ou... = No

Skip To: Q152 If Did you attend other appointments? (e.g., alternative therapies, private scans that you sought ou... = Not applicable

Skip To: Q152 If Did you attend other appointments? (e.g., alternative therapies, private scans that you sought ou... = I don't know/ I can't remember

Please specify the other types of appointments you attended and how many times you attended?

|  | Did not attend (4) | Once (1) | 2-3 times (2) | More than 3 times (3) | Not applicable (5) |
| --- | --- | --- | --- | --- | --- |
| Private scan (4) |  |  |  |  |  |
| Urgent care (Emergency Department) (5) |  |  |  |  |  |
| Out of hours family doctor service (6) |  |  |  |  |  |
| Alternative Therapies (e.g., Yoga, homoeopathy, hypnosis acupuncture) (7) |  |  |  |  |  |
| Physiotherapy (10) |  |  |  |  |  |
| Fertility services (9) |  |  |  |  |  |
| Other (8) |  |  |  |  |  |

| Page Break |  |
| --- | --- |

**Section 8: Overall Care**
 In this section, we want to find out about your **overall care in relation to recurrent miscarriage services and supports**. When answering these questions, think about the time periods covered in the sections above. For example, **if you were investigated more than once (and/or had a subsequent loss), please answer the question based on when you were first investigated for recurrent miscarriage.**

Overall, how would you rate your experience of the care you received for recurrent miscarriage? 


[Select one from one to ten, where one is a very poor experience and ten is a very good experience]

|  | I had a very poor experience 1 (1) | 2 (2) | 3 (3) | 4 (4) | 5 (5) | 6 (6) | 7 (7) | 8 (8) | 9 (9) | I had a very good experience 10 (10) |
| --- | --- | --- | --- | --- | --- | --- | --- | --- | --- | --- |
| Your experience of the care you received for recurrent miscarriage (1) |  |  |  |  |  |  |  |  |  |  |

| Page Break |  |
| --- | --- |

Overall, thinking about the care you received for recurrent miscarriage, was it?

- Much better than I expected (1)
- Somewhat better than I expected (2)
- Exactly as I expected (3)
- Somewhat worse than I expected (4)
- Much worse than I expected (5)

| Page Break |  |
| --- | --- |

Overall, since you were diagnosed with recurrent miscarriage, have staff in different places worked well together when caring for you (e.g. information about you passed on, no unnecessary delays)?

- Yes (1)
- To some extent (2)
- No, not really (3)

| Page Break |  |
| --- | --- |

**Work experience and recurrent miscarriage**
 We would like you to describe your work experiences during your recurrent miscarriage care (investigations/ treatment). For each of the following statements, please indicate your agreement or disagreement with the statements.
 
I went to work, but because of receiving recurrent miscarriage care (investigations, treatment/care plan, and/or support services):

|  | Strongly disagree (1) | Somewhat disagree (2) | Uncertain (3) | Somewhat agree (4) | Strongly agree (5) | Not applicable (6) |
| --- | --- | --- | --- | --- | --- | --- |
| The stresses of my job were much harder to handle (1) |  |  |  |  |  |  |
| I was able to finish hard tasks in my work. (2) |  |  |  |  |  |  |
| I was distracted from taking pleasure in my work (3) |  |  |  |  |  |  |
| I felt hopeless about finishing certain work tasks (4) |  |  |  |  |  |  |
| I was able to focus on achieving my goals (5) |  |  |  |  |  |  |
| I felt energetic enough to complete all my work. (6) |  |  |  |  |  |  |

| Page Break |  |
| --- | --- |

**Section 9: Your Health and Well-Being**
 
This survey asks for your views about your health. This information will help keep track of how you feel and how well you are able to do your usual activities. Thank you for completing this survey!
 
For each of the following questions, please select the one response that best describes your answer. 

In general, would you say your health is:

- Excellent (6)
- Very good (7)
- Good (8)
- Fair (9)
- Poor (10)

| Page Break |  |
| --- | --- |

The following questions are about activities you might do during a typical day. 
 
Does your health now limit you in these activities? If so, how much

Does your health now limit you in moderate activities such as moving a table, pushing a vacuum cleaner, bowling, or playing golf? If so, how much?

- Yes, limited a lot (1)
- Yes, limited a little (2)
- No, not limited at all (3)

Does your health now limit you in climbing several flights of stairs? If so, how much?

- Yes, limited a lot (1)
- Yes, limited a little (2)
- No, not limited at all (3)

| Page Break |  |
| --- | --- |

During the past week, how much of the time have you had any of the following problems with your work or other regular daily activities as a result of your physical health?

During the past week, how much of the time have you accomplished less than you would have liked as a result of your physical health?

- All of the time (1)
- Most of the time (2)
- Some of the time (3)
- A little of the time (4)
- None of the time (5)

During the past week, how much of the time were you limited in the kind of work or other activities you do as a result of your physical health?

- All of the time (1)
- Most of the time (2)
- Some of the time (3)
- A little of the time (4)
- None of the time (5)

| Page Break |  |
| --- | --- |

During the past week, how much of the time have you had any of the following problems with your work or other regular daily activities as a result of any emotional problems (such as feeling depressed or anxious)?

During the past week, how much of the time have you accomplished less than you would have liked as a result of any emotional problems (such as feeling depressed or anxious)?

- All of the time (1)
- Most of the time (2)
- Some of the time (3)
- A little of the time (4)
- None of the time (5)

During the past week, how much of the time did you do work or other activities less carefully than usual as a result of any emotional problems (such as feeling depressed or anxious)?

- All of the time (1)
- Most of the time (2)
- Some of the time (3)
- All of the time (4)
- None of the time (5)

| Page Break |  |
| --- | --- |

During the past week, how much did pain interfere with your normal work (including both work outside the home and housework)?

- Not at al (1)
- Slightly (2)
- Moderately (3)
- Quite a bit (4)
- Extremely (5)

| Page Break |  |
| --- | --- |

These questions are about how you feel and how things have been with you during the past week. For each question, please give the one answer that comes closest to the way you have been feeling.

How much of the time during the past week have you felt calm and peaceful?

- All of the time (1)
- Most of the time (2)
- Some of the time (3)
- A little of the time (4)
- None of the time (5)

How much of the time during the past week did you have a lot of energy?

- All of the time (1)
- Most of the time (2)
- Some of the time (3)
- A little of the time (4)
- None of the time (5)

How much of the time during the past week have you felt downhearted and low?

- All of the time (1)
- Most of the time (2)
- Some of the time (3)
- A little of the time (4)
- None of the time (5)

| Page Break |  |
| --- | --- |

During the past week, how much of the time has your physical health or emotional problems interfered with your social activities (like visiting with friends, relatives, etc.)?

- All of the time (1)
- Most of the time (2)
- Some of the time (3)
- A little of the time (4)
- None of the time (5)

| Page Break |  |
| --- | --- |

**Section 10: Value placed on recurrent miscarriage services**
 You may have r**eceived or are receiving care for recurrent miscarriag**e: We want to know the **financial amount you would place on the value you get/could get from receiving recurrent miscarriage care/services.** This will help us to complete an evaluation of the costs and benefits of the services provided.

When receiving recurrent miscarriage care, did you attend a dedicated recurrent miscarriage clinic? 
 
*(e.g., a dedicated recurrent miscarriage clinic is an organised model of care which provides dedicated consultant-led clinics. The dedicated clinic is supported by maternity unit/hospital departments and includes access to investigations, treatment plan, and specialist counselling. This clinic coordinates all your recurrent miscarriage care, including investigations, management, treatment, and access to supportive services)*

- Yes (1)
- No (2)
- I don’t know / I can’t remember (3)

Skip To: Q168 If When receiving recurrent miscarriage care, did you attend a dedicated recurrent miscarriage clini... = No

Skip To: Q168 If When receiving recurrent miscarriage care, did you attend a dedicated recurrent miscarriage clini... = I don’t know / I can’t remember

| Page Break |  |
| --- | --- |

Display This Question:

If When receiving recurrent miscarriage care, did you attend a dedicated recurrent miscarriage clini... = Yes

Please specify the name of the maternity unit/hospital where this recurrent miscarriage clinic was/is based?

________________________________________________________________

Skip To: Q260 If Condition: Please specify the name of ... Is Displayed. Skip to: Overall, how far would you travel to ....

| Page Break |  |
| --- | --- |

Would you attend a dedicated recurrent miscarriage clinic based in your local/nearest maternity hospital/unit?

- Yes (1)
- No (2)
- Maybe (3)

Skip To: Q170 If Would you attend a dedicated recurrent miscarriage clinic based in your local/nearest maternity h... = No

| Page Break |  |
| --- | --- |

Overall, how far would you travel to attend a dedicated recurrent miscarriage clinic?

- <30 minutes (1)
- 30-1 hours (2)
- 1hr -2 hours (3)
- >2 hours (4)

| Page Break |  |
| --- | --- |

**Recurrent miscarriage care is free, and it will remain free (unless you opt for private care).**

 
In this section, we want to find out **how strongly you feel about having a dedicated recurrent miscarriage clinic in your maternity hospital/unit** [whether there is one in the hospital you attend/not]. 
 
**So, imagine that you do have to pay. There are no right or wrong answers.**
 
(Please write your answer in €. One way to think of this is to imagine you are at an auction at which the most you would pay for an item shows the importance you place on that item. How far are you prepared to go?)
 
 
This dedicated recurrent miscarriage clinic would provide your recurrent miscarriage care including consultant visits, investigations, treatments, and access to bereavement specialists, such as midwives, social workers, and counsellors. Plus, supportive services for subsequent pregnancy (access to early scans and counselling) and referrals to additional services if indicated.

Carry Forward All Choices – Displayed & Hidden from "Hypothetically, what is the maximum amount of money you would be prepared to pay to attend this clinic after you had your second consecutive miscarriage?"

| 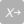 |
| --- |

Hypothetically, what is the maximum amount of money you would be prepared to pay to attend this clinic after you had your second consecutive miscarriage?

▼ < €500 (1) ... > €7,000 (9)

Carry Forward All Choices – Displayed & Hidden from "Hypothetically, what is the maximum amount of money you would be prepared to pay to attend this clinic after you had your second consecutive miscarriage?"

| 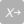 |
| --- |

Hypothetically, what is the maximum amount of money you would be prepared to pay to attend this clinic after/if you had three or more consecutive miscarriages?

▼ < €500 (1) ... > €7,000 (9)

| Page Break |  |
| --- | --- |

End of Block: Main body

Start of Block: Partner

Display This Question:

If Which best describes you? = Partner / Father

**Section 2 Partners experience of recurrent miscarriage care**
In this section, we will ask you about your view/perceptions of the care you received and experienced for recurrent miscarriage. If you/your partner have been investigated/treated multiple times for recurrent miscarriage, we ask that you think about **the first time that you had investigations/treatment.**

Display This Question:

If Which best describes you? = Partner / Father

How many years ago were you/ your partner first referred for investigation for recurrent miscarriage? [Please enter number of years below]

________________________________________________________________

| Page Break |  |
| --- | --- |

Display This Question:

If Which best describes you? = Partner / Father

Carry Forward All Choices – Displayed & Hidden from "Did your investigations for recurrent miscarriage take place in any of the following locations:"

| 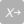 |
| --- |

Was your care for recurrent miscarriage carried out in any of the following locations?

|  | Yes (1) | No (2) | I don't know/ I can't remember (3) |
| --- | --- | --- | --- |
| General practice (GP/Practice Nurse) (x1) |  |  |  |
| Hospital-Early Pregnancy Unit (x2) |  |  |  |
| Hospital-pregnancy loss clinic (x3) |  |  |  |
| Hospital-recurrent miscarriage clinic (x4) |  |  |  |
| Hospital-gynaecology clinic (x5) |  |  |  |
| Phlebotomy clinic (x6) |  |  |  |
| Private consultant rooms (x7) |  |  |  |
| Fertility clinic (x8) |  |  |  |
| Other. Please specify: (x9) |  |  |  |

| Page Break |  |
| --- | --- |

Display This Question:

If Which best describes you? = Partner / Father

Did you attend any of the following appointments?

|  | Yes (1) | No (10) | Not facilitated due to COVID-19 restrictions (11) | I don’t know/ I can’t remember (12) |
| --- | --- | --- | --- | --- |
| **Investigation appointments** (Investigation appointments refer to any appointments which involved undergoing medical tests to determine a cause for your recurrent miscarriage) (1) |  |  |  |  |
| **Receiving investigation results** (Appointments in which results from recurrent miscarriage investigations were received) (5) |  |  |  |  |
| **Treatment/plan of care appointments** (Appointments where a plan was put in place for a future pregnancy following initial investigations for recurrent miscarriage) (6) |  |  |  |  |
| **Subsequent pregnancy care appointments** (Appointments for care received if your partner got pregnant again such as reassurance scans) (12) |  |  |  |  |

| Page Break |  |
| --- | --- |

Display This Question:

If Which best describes you? = Partner / Father

Were you told who to contact if you had questions/concerns for each of the following:

|  | Yes (1) | No (4) | I don’t know/ I can’t remember (5) |
| --- | --- | --- | --- |
| **Investigations/ medical tests** (1) |  |  |  |
| **Results of investigations** (4) |  |  |  |
| **Treatment/plan of care** (5) |  |  |  |
| **Subsequent pregnancy care** (6) |  |  |  |

| Page Break |  |
| --- | --- |

Display This Question:

If Which best describes you? = Partner / Father

Were you involved as much as you wanted to be in decisions for each of the following:

|  | Yes, definitely (1) | Yes, to some extent (4) | No (5) | I don’t know/ I can’t remember (6) | Not applicable (7) |
| --- | --- | --- | --- | --- | --- |
| **Investigations/ medical tests** (10) |  |  |  |  |  |
| **Treatment/ plan of care** (8) |  |  |  |  |  |
| **Subsequent pregnancy care** (9) |  |  |  |  |  |

| Page Break |  |
| --- | --- |

Display This Question:

If Which best describes you? = Partner / Father

Carry Forward All Choices – Displayed & Hidden from "Were you told who to contact if you had questions/concerns for each of the following:"

| 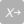 |
| --- |

Did you receive enough information for each of the following:

|  | Yes, definitely (1) | Yes, to some extent (4) | No (5) | I don’t know/ I can’t remember (6) | Not applicable (7) |
| --- | --- | --- | --- | --- | --- |
| **Investigations/ medical tests** (x1) |  |  |  |  |  |
| **Results of investigations** (x4) |  |  |  |  |  |
| **Treatment/plan of care** (x5) |  |  |  |  |  |
| **Subsequent pregnancy care** (x6) |  |  |  |  |  |

| Page Break |  |
| --- | --- |

Display This Question:

If Which best describes you? = Partner / Father

Carry Forward All Choices – Displayed & Hidden from "Did you receive enough information for each of the following:"

| 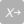 |
| --- |

When you had questions, did you get answers that you could understand for each of the following:

|  | Yes, always (1) | Yes, sometimes, (4) | No (5) | I did not have the opportunity to ask questions (6) | I did not need to ask questions (7) | I don’t know/ I can’t remember (8) | Not applicable (9) |
| --- | --- | --- | --- | --- | --- | --- | --- |
| **Investigations/ medical tests** (xx1) |  |  |  |  |  |  |  |
| **Results of investigations** (xx4) |  |  |  |  |  |  |  |
| **Treatment/plan of care** (xx5) |  |  |  |  |  |  |  |
| **Subsequent pregnancy care** (xx6) |  |  |  |  |  |  |  |

| Page Break |  |
| --- | --- |

Display This Question:

If Which best describes you? = Partner / Father

Carry Forward All Choices – Displayed & Hidden from "When you had questions, did you get answers that you could understand for each of the following:"

| 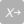 |
| --- |

Did you have a healthcare professional that you could talk to about your worries and fears for each of the following:

|  | Yes, always (1) | Yes, sometimes, (4) | No (5) | I had no worries or fears (6) | I don’t know/ I can’t remember (7) | Not applicable (8) |
| --- | --- | --- | --- | --- | --- | --- |
| **Investigations/ medical tests** (xxx1) |  |  |  |  |  |  |
| **Results of investigations** (xxx4) |  |  |  |  |  |  |
| **Treatment/plan of care** (xxx5) |  |  |  |  |  |  |
| **Subsequent pregnancy care** (xxx6) |  |  |  |  |  |  |

| Page Break |  |
| --- | --- |

Display This Question:

If Which best describes you? = Partner / Father

Carry Forward All Choices – Displayed & Hidden from "Did you have a healthcare professional that you could talk to about your worries and fears for each of the following:"

| 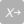 |
| --- |

Did you have confidence and trust in those providing your care for each of the following:

|  | Yes, always (1) | Yes, sometimes (4) | No (5) | I don’t know/ I can’t remember (6) | Not applicable (7) |
| --- | --- | --- | --- | --- | --- |
| **Investigations/ medical tests** (xxxx1) |  |  |  |  |  |
| **Results of investigations** (xxxx4) |  |  |  |  |  |
| **Treatment/plan of care** (xxxx5) |  |  |  |  |  |
| **Subsequent pregnancy care** (xxxx6) |  |  |  |  |  |

| Page Break |  |
| --- | --- |

Display This Question:

If Which best describes you? = Partner / Father

Carry Forward All Choices – Displayed & Hidden from "Did you have confidence and trust in those providing your care for each of the following:"

| 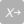 |
| --- |

Did it ever happen that one healthcare professional said one thing, and another said something different for each of the following:

|  | Often (1) | Sometimes (4) | Only once (5) | Never (6) | I don't know / I can't remember (3) | Not applicable (7) |
| --- | --- | --- | --- | --- | --- | --- |
| **Investigations/ medical tests** (xxxxx1) |  |  |  |  |  |  |
| **Results of investigations** (xxxxx4) |  |  |  |  |  |  |
| **Treatment/plan of care** (xxxxx5) |  |  |  |  |  |  |
| **Subsequent pregnancy care** (xxxxx6) |  |  |  |  |  |  |

| Page Break |  |
| --- | --- |

Display This Question:

If Which best describes you? = Partner / Father

Carry Forward All Choices – Displayed & Hidden from "Did it ever happen that one healthcare professional said one thing, and another said something different for each of the following:"

| 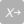 |
| --- |

Did you ever think that the healthcare professionals were deliberately not telling you certain things that you wanted to know for each of the following?

|  | Often (1) | Sometimes (4) | Only once (5) | Never (6) | I don’t know/ I can’t remember (7) | Not applicable (8) |
| --- | --- | --- | --- | --- | --- | --- |
| **Investigations/ medical tests** (xxxxxx1) |  |  |  |  |  |  |
| **Results of investigations** (xxxxxx4) |  |  |  |  |  |  |
| **Treatment/plan of care** (xxxxxx5) |  |  |  |  |  |  |
| **Subsequent pregnancy care** (xxxxxx6) |  |  |  |  |  |  |

| Page Break |  |
| --- | --- |

Display This Question:

If Which best describes you? = Partner / Father

Carry Forward All Choices – Displayed & Hidden from "Did you ever think that the healthcare professionals were deliberately not telling you certain things that you wanted to know for each of the following?"

| 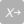 |
| --- |

Do you think the healthcare professionals involved did everything they could for you during each of the following stages:

|  | Yes, definitely (1) | Yes, to some extent (4) | No (5) | I don’t know/ I can’t remember (6) | Not applicable (7) |
| --- | --- | --- | --- | --- | --- |
| **Investigations/ medical tests** (xxxxxxx1) |  |  |  |  |  |
| **Results of investigations** (xxxxxxx4) |  |  |  |  |  |
| **Treatment/plan of care** (xxxxxxx5) |  |  |  |  |  |
| **Subsequent pregnancy care** (xxxxxxx6) |  |  |  |  |  |

| Page Break |  |
| --- | --- |

Display This Question:

If Which best describes you? = Partner / Father

Carry Forward All Choices – Displayed & Hidden from "Did you ever think that the healthcare professionals were deliberately not telling you certain things that you wanted to know for each of the following?"

| 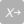 |
| --- |

Did you feel you were treated with respect and dignity for each of the following:

|  | Yes, always (1) | Yes, sometimes (4) | No (5) | I don’t know / I can’t remember (6) | Not applicable (7) |
| --- | --- | --- | --- | --- | --- |
| **Investigations/ medical tests** (xxxxxxx1) |  |  |  |  |  |
| **Results of investigations** (xxxxxxx4) |  |  |  |  |  |
| **Treatment/plan of care** (xxxxxxx5) |  |  |  |  |  |
| **Subsequent pregnancy care** (xxxxxxx6) |  |  |  |  |  |

| Page Break |  |
| --- | --- |

Display This Question:

If Which best describes you? = Partner / Father

**Section 3: Information and Support Services**
In this section, we will ask you about your experience of information and support services for recurrent miscarriage.

Display This Question:

If Which best describes you? = Partner / Father

Were you told about/given information about any of the following supports for recurrent miscarriage?

|  | Yes (1) | No (2) | I don't know/ I can't remember (3) |
| --- | --- | --- | --- |
| Clinical Midwife/Nurse Specialist in Bereavement and Loss (1) |  |  |  |
| Miscarriage Association of Ireland (2) |  |  |  |
| Féileacáin (Stillbirth and Neonatal Death Association of Ireland) (3) |  |  |  |
| Online discussion boards/forums (4) |  |  |  |
| Chaplaincy and/or pastoral care (5) |  |  |  |
| Religious or spiritual support group (6) |  |  |  |
| One-on-one counselling (i.e. - with a Therapist, Counsellor, Psychologist) (7) |  |  |  |
| Psychiatry (8) |  |  |  |
| Social work service (9) |  |  |  |
| Perinatal mental health (10) |  |  |  |
| Books/booklets/leaflets/pamphlets (11) |  |  |  |
| Pregnancy and Infant Loss website www.pregnancyandinfantloss.ie (12) |  |  |  |
| Cork Miscarriage website www.corkmiscarriage.com (13) |  |  |  |
| Other. Please specify: (14) |  |  |  |

| Page Break |  |
| --- | --- |

Display This Question:

If Which best describes you? = Partner / Father

If you used any of the following supports, please indicate how helpful they have been. [Please select 'did not use' if you did not access any of the following]

|  | Extremely helpful (1) | Very helpful (2) | Somewhat helpful (3) | Not very helpful (4) | Not at all helpful (5) | Not sure (6) | Did not use (7) |
| --- | --- | --- | --- | --- | --- | --- | --- |
| Clinical Midwife/Nurse Specialist in Bereavement and Loss (1) |  |  |  |  |  |  |  |
| Miscarriage Association of Ireland (2) |  |  |  |  |  |  |  |
| Féileacáin (Stillbirth and Neonatal Death Association of Ireland) (3) |  |  |  |  |  |  |  |
| Online discussion boards/forums (4) |  |  |  |  |  |  |  |
| Chaplaincy and/or pastoral care (5) |  |  |  |  |  |  |  |
| Religious or spiritual support group (6) |  |  |  |  |  |  |  |
| One-on-one counselling (i.e. - with a Therapist, Counsellor, Psychologist) (7) |  |  |  |  |  |  |  |
| Psychiatry (8) |  |  |  |  |  |  |  |
| Social work service (9) |  |  |  |  |  |  |  |
| Perinatal mental health (10) |  |  |  |  |  |  |  |
| Books/booklets/leaflets/pamphlets (11) |  |  |  |  |  |  |  |
| Pregnancy and Infant Loss website www.pregnancyandinfantloss.ie (12) |  |  |  |  |  |  |  |
| Cork Miscarriage website www.corkmiscarriage.com (13) |  |  |  |  |  |  |  |
| Other (14) |  |  |  |  |  |  |  |

| Page Break |  |
| --- | --- |

Display This Question:

If Which best describes you? = Partner / Father

**Section 4: Overall Care**
 In this section, we want to find out about **your overall care** in relation to recurrent miscarriage services and supports. When answering these questions, think about the time period covered in the section above. For example, if you/ your partner have been investigated/treated multiple times for recurrent miscarriage, we ask that you think about the **first time that you had investigations/treatment.**

Display This Question:

If Which best describes you? = Partner / Father

Overall, how would you rate your experience of the care you/ your partner received for recurrent miscarriage? 


[Select one from one to ten, where one is a very poor experience and ten is a very good experience]

|  | I had a very poor experience 1 (1) | 2 (2) | 3 (3) | 4 (4) | 5 (5) | 6 (6) | 7 (7) | 8 (8) | 9 (9) | I had a very good experience 10 (10) |
| --- | --- | --- | --- | --- | --- | --- | --- | --- | --- | --- |
| Your experience of the care you received for recurrent miscarriage (1) |  |  |  |  |  |  |  |  |  |  |

| Page Break |  |
| --- | --- |

Display This Question:

If Which best describes you? = Partner / Father

Overall, thinking about the care you/ your partner received for recurrent miscarriage, was it?

- Much better than I expected (1)
- Somewhat better than I expected (2)
- Exactly as I expected (3)
- Somewhat worse than I expected (4)
- Much worse than I expected (5)

| Page Break |  |
| --- | --- |

Display This Question:

If Which best describes you? = Partner / Father

Overall, since you/ your partner were diagnosed with recurrent miscarriage, have staff in different places worked well together when caring for you (e.g. information about you/ your was passed on, no unnecessary delays)?

- Yes (1)
- To some extent (2)
- No, not really (3)

| Page Break |  |
| --- | --- |

Display This Question:

If Which best describes you? = Partner / Father

**Work experience and recurrent miscarriage**
 We would like you to describe your work experiences during your recurrent miscarriage care. For each of the following statements, please indicate your agreement or disagreement with the statements.
 
I went to work, but because of receiving recurrent miscarriage care (investigations, treatment/care plan, and/or support services):

|  | Strongly disagree (1) | Somewhat disagree (2) | Uncertain (3) | Somewhat agree (4) | Strongly agree (5) | Not applicable (6) |
| --- | --- | --- | --- | --- | --- | --- |
| The stresses of my job were much harder to handle (1) |  |  |  |  |  |  |
| I was able to finish hard tasks in my work. (2) |  |  |  |  |  |  |
| I was distracted from taking pleasure in my work (3) |  |  |  |  |  |  |
| I felt hopeless about finishing certain work tasks (4) |  |  |  |  |  |  |
| I was able to focus on achieving my goals (5) |  |  |  |  |  |  |
| I felt energetic enough to complete all my work. (6) |  |  |  |  |  |  |

| Page Break |  |
| --- | --- |

Display This Question:

If Which best describes you? = Partner / Father

**Section 5: Your Health and Well-Being**
This section asks for your views about your health. This information will help keep track of how you feel and how well you are able to do your usual activities. 
 

 For each of the following questions, please select the one response that best describes your answer.

Display This Question:

If Which best describes you? = Partner / Father

In general, would you say your health is:

- Excellent (1)
- Very good (2)
- Good (3)
- Fair (4)
- Poor (5)

| Page Break |  |
| --- | --- |

Display This Question:

If Which best describes you? = Partner / Father

The following questions are about activities you might do during a typical day.

 Does your health now limit you in these activities? If so, how much?

Display This Question:

If Which best describes you? = Partner / Father

Does your health now limit you in moderate activities, such as moving a table, pushing a vacuum cleaner, bowling, or playing golf? If so, how much?

- Yes, limited a lot (1)
- Yes, limited a little (2)
- No, not limited at all (3)

Display This Question:

If Which best describes you? = Partner / Father

Does your health now limit you in climbing several flights of stairs? If so, how much?

- Yes, limited a lot (1)
- Yes, limited a little (2)
- No, not limited at all (3)

| Page Break |  |
| --- | --- |

Display This Question:

If Which best describes you? = Partner / Father

During the past week, how much of the time have you had any of the following problems with your work or other regular daily activities as a result of your physical health?

Display This Question:

If Which best describes you? = Partner / Father

During the past week, how much of the time have you accomplished less than you would have liked as a result of your physical health?

- All of the time (1)
- Most of the time (2)
- Some of the time (3)
- A little of the time (4)
- None of the time (5)

Display This Question:

If Which best describes you? = Partner / Father

During the past week, how much of the time were you limited in the kind of work or other activities you do as a result of your physical health?

- All of the time (1)
- Most of the time (2)
- Some of the time (3)
- A little of the time (4)
- None of the time (5)

| Page Break |  |
| --- | --- |

Display This Question:

If Which best describes you? = Partner / Father

During the past week, how much of the time have you had any of the following problems with your work or other regular daily activities as a result of any emotional problems (such as feeling depressed or anxious)?

Display This Question:

If Which best describes you? = Partner / Father

During the past week, how much of the time have you accomplished less than you would have liked as a result of any emotional problems (such as feeling depressed or anxious)?

- All of the time (1)
- Most of the time (2)
- Some of the time (3)
- A little of the time (4)
- None of the time (5)

Display This Question:

If Which best describes you? = Partner / Father

During the past week, how much of the time did you do work or other activities less carefully than usual as a result of any emotional problems (such as feeling depressed or anxious)?

- All of the time (1)
- Most of the time (2)
- Some of the time (3)
- A little of the time (4)
- None of the time (5)

| Page Break |  |
| --- | --- |

Display This Question:

If Which best describes you? = Partner / Father

During the past week, how much did pain interfere with your normal work (including work both outside the home and housework)?

- Not at all (1)
- Slightly (2)
- Moderately (3)
- Quite a bit (4)
- Extremely (5)

| Page Break |  |
| --- | --- |

Display This Question:

If Which best describes you? = Partner / Father

These questions are about how you feel and how things have been with you during the past week. For each question, please give the one answer that comes closest to the way you have been feeling.

Display This Question:

If Which best describes you? = Partner / Father

How much of the time during the past week have you felt calm and peaceful?

- All of the time (1)
- Most of the time (2)
- Some of the time (3)
- A little of the time (4)
- None of the time (5)

Display This Question:

If Which best describes you? = Partner / Father

How much of the time during the past week did you have a lot of energy?

- All of the time (1)
- Most of the time (2)
- Some of the time (3)
- A little of the time (4)
- None of the time (5)

Display This Question:

If Which best describes you? = Partner / Father

How much of the time during the past week have you felt downhearted and low?

- All of the time (1)
- Most of the time (2)
- Some of the time (3)
- A little of the time (4)
- None of the time (5)

| Page Break |  |
| --- | --- |

Display This Question:

If Which best describes you? = Partner / Father

During the past week, how much of the time has your physical health or emotional problems interfered with your social activities (like visiting friends, relatives, etc.)?

- All of the time (1)
- Most of the time (2)
- Some of the time (3)
- A little of the time (4)
- None of the time (5)

| Page Break |  |
| --- | --- |

Display This Question:

If Which best describes you? = Partner / Father

**Section 6: Value placed on recurrent miscarriage services**
 **You may have received or are receiving care for recurrent miscarriage**. We want to know the **financial amount** you would **place on the value you get/could get from receiving recurrent miscarriage care/services**. This will help us to complete an evaluation of the costs and benefits of the services provided.

Display This Question:

If Which best describes you? = Partner / Father

When receiving recurrent miscarriage care, did you/ your partner attend a dedicated recurrent miscarriage clinic? 
 
*(e.g., a dedicated recurrent miscarriage clinic is an organised model of care which provides dedicated consultant-led clinics. The dedicated clinic is supported by maternity unit/hospital departments and includes access to investigations, treatment plan, and specialist counselling. This clinic coordinates all your recurrent miscarriage care, including investigations, management, treatment, and access to supportive services)*

- Yes (1)
- No (2)
- I don’t know / I can’t remember (3)

Skip To: Q259 If When receiving recurrent miscarriage care, did you/ your partner attend a dedicated recurrent mis... = No

Skip To: Q259 If When receiving recurrent miscarriage care, did you/ your partner attend a dedicated recurrent mis... = I don’t know / I can’t remember

| Page Break |  |
| --- | --- |

Display This Question:

If Which best describes you? = Partner / Father

Please specify the name of the maternity unit/hospital where this recurrent miscarriage clinic was/is based?

________________________________________________________________

Skip To: Q169 If Condition: Please specify the name of ... Is Displayed. Skip to: Overall, how far would you travel to ....

| Page Break |  |
| --- | --- |

Display This Question:

If Which best describes you? = Partner / Father

Would you/ your partner attend a dedicated recurrent miscarriage clinic based in your local/nearest maternity hospital/unit?

- Yes (1)
- No (2)
- Maybe (3)

Skip To: Q261 If Would you/ your partner attend a dedicated recurrent miscarriage clinic based in your local/neare... = No

| Page Break |  |
| --- | --- |

Display This Question:

If Which best describes you? = Partner / Father

Overall, how far would you travel to attend a dedicated recurrent miscarriage clinic?

- <30 minutes (1)
- 30-1 hours (2)
- 1hr -2 hours (3)
- >2 hours (4)

| Page Break |  |
| --- | --- |

Display This Question:

If Which best describes you? = Partner / Father

**Recurrent miscarriage care is free, and it will remain free (unless you opt for private care).**

 
In this section, we want to find out **how strongly you feel about having a dedicated recurrent miscarriage clinic in your maternity hospital/unit** [whether there is one in the hospital you attend or not]. 
 
So, imagine that you do have to pay. There are no right or wrong answers. 
 
(Please select from the drop-down list the amount you are willing to pay. One way to think of this is to imagine you are at an auction at which the most you would pay for an item shows the importance you place on that item. How far are you prepared to go?)
 
 
This dedicated recurrent miscarriage clinic would provide your recurrent miscarriage care including consultant visits, investigations, treatments, and access to bereavement specialists, such as midwives, social workers, and counsellors. Plus, supportive services for subsequent pregnancy (access to early scans and counselling) and referrals to additional services if indicated.

Display This Question:

If Which best describes you? = Partner / Father

Hypothetically, what is the maximum amount of money you would be prepared to pay to attend this clinic after you had your second consecutive miscarriage?

▼ < €500 (1) ... > €7,000 (7)

Display This Question:

If Which best describes you? = Partner / Father

Carry Forward All Choices – Displayed & Hidden from "Hypothetically, what is the maximum amount of money you would be prepared to pay to attend this clinic after you had your second consecutive miscarriage?"

| 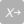 |
| --- |

Hypothetically, what is the maximum amount of money you would be prepared to pay to attend this clinic after/if you had three or more consecutive miscarriages?

▼ < €500 (1) ... > €7,000 (9)

End of Block: Partner

Start of Block: Any Other Comments

**Any other comments**

Were there any other important parts of your care experience that are not covered by the questions in this survey that you would like to tell us about? If so, please write them here:

________________________________________________________________

| Page Break |  |
| --- | --- |

**Thank you for taking the time to complete this survey.**

 The survey will provide insight into the care provided for recurrent miscarriage and will identify the types of services and supports that may help at such a time. The results will be used to improve existing services and supports.

 **Your response has been recorded, you can now leave the survey.**

 **If you completing this survey has caused you distress, please talk to your care provider or contact the support services below:**

 [Pregnancy and Infant Loss Website](https://pregnancyandinfantloss.ie/)
 [The Miscarriage Association of Ireland](http://www.miscarriage.ie/) provide peer to peer support to those who have experienced miscarriage
 [Féileacáin](https://feileacain.ie/)provide peer to peer support to bereaved parents 
 [A Little Lifetime](https://alittlelifetime.ie/) provide peer to peer support to bereaved parents
 [Cork Miscarriage](http://www.corkmiscarriage.com) Information and Resources 


 We will not be sharing feedback with individual hospitals. If you would like to make a complaint about the care you received for recurrent miscarriage you can find more information at [Citizens Information](https://www.citizensinformation.ie/en/health/health_system/making_a_complaint_about_the_health_service_executive.html).  

 For information about making a complaint about a private health service, visit [Health Complaints](https://www.healthcomplaints.ie/), a website that provides information on making complaints about private and public health services.

 There is an HSE complaints system for anyone seeking or getting public health or social care services provided by the HSE. The complaints system also covers service providers who provide health or social care services on behalf of the HSE. If you want to make a complaint about your experience in a public hospital, [Patient Advocacy Service (PAS)](https://www.patientadvocacyservice.ie/) can provide information and support. The service is independent, free and confidential and it applies to public acute hospitals that are funded by the HSE.

 **If you have any questions regarding this study or if you have any concerns related to your involvement, please contact the research team:** Marita Hennessy, PhD,  Postdoctoral Researcher, RE:CURRENT Project, [maritahennessy@ucc.ie](mailto:maritahennessy@ucc.ie), or Caragh Flannery, PhD, Postdoctoral Researcher, RE:CURRENT Project [cflannery@ucc.ie](mailto:cflannery@ucc.ie)

SF-12v2® Health Survey Ó 1992, 2002, 2013 Medical Outcomes Trust and QualityMetric Incorporated.
 All rights reserved.
 SF-12® is a registered trademark of Medical Outcomes Trust. 
(SF-12v2® Health Survey Acute, United Kingdom (English))

End of Block: Any Other Comments
