# Supplementary material for: Factors that shape recurrent miscarriage care experiences: findings from a national survey
Source: BMC Health Serv Res. 2023 Mar 31;23:317. doi: 10.1186/s12913-023-09347-1 (PMC10064661; doi:10.1186/s12913-023-09347-1)
Supplement: Supplementary file 2 — Additional file 2: Table S2.1. Men’s characteristics. Figure S2.1. Location where the majority of RM investigations were carried out (Men). Figure S2.2. Attendance at RM care appointments (Men). Table S2.2. Patient-centred care items for investigation, receiving results, treatment/plan of care, and subsequent pregnancy (Men). Figure S2.3. RM care experience (Men). [file 12913_2023_9347_MOESM2_ESM.docx]

**Additional File 2.**

**Table S2.1. Men’s characteristics (n=4)**

| **Variable (n=135)** | **N (%)** |
| --- | --- |
| **Age** |  |
| 24-34 years | 1 (25) |
| 35-44 years | 1 (25) |
| 55-64 years | 2 (50) |
| **Cultural background** |  |
| White – Irish | 4 (100) |
| **Nationality** |  |
| Irish | 4 (100) |
| **Relationship status** |  |
| Married | 4 (100) |
| **Education level** |  |
| Secondary school or less | 1 (25) |
| University degree | 2 (50) |
| Postgraduate Degree (Masters or PhD) | 1 (25) |
| **Employment** |  |
| Employed full-time | 3 (75) |
| Employed part-time | 1 (25) |
| **Medical Cover** |  |
| Private health insurance holder | 2 (50) |
| None | 2 (50) |
| **Number of consecutive losses experienced** |  |
| Two | 1 (25) |
| Three | 1 (25) |
| Four or more | 2 (50) |
| **Year RM care first received** |  |
| 2011-2015 | 2 (50) |
| 2016-2021 | 2 (50) |
| **Diagnosed with infertility** |  |
| No | 4 (100) |

**Figure S2.1. Location where the majority of RM investigations were carried out (Men, n=4)**

*PLC, pregnancy loss clinic, RMC, recurrent miscarriage clinic; EPAU, early pregnancy assessment unit; GP, general practitioner*


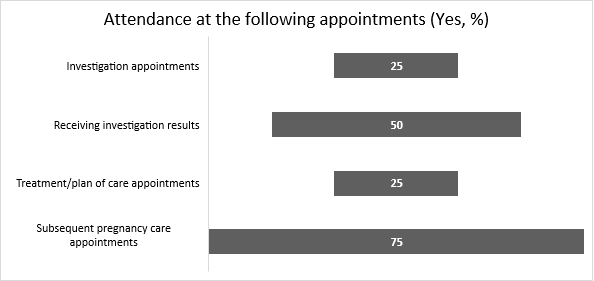


**Figure S2.2. Attendance at RM care appointments (Men, n=4)**

**Table S2.2. Patient-centred care items for investigation, receiving results, treatment/plan of care, and subsequent pregnancy (Men, n=4)**

| **Variable** | **Investigations** | **Receiving results** | **Treatment/ plan of care** | **Subsequent pregnancy** |
| --- | --- | --- | --- | --- |
| **Involved as much as you wanted to be in decisions** |  |  |  |  |
| No | 2 (50) | 1 (25) | - | 1 (25) |
| Yes | 2 (50) | 3 (75) | - | 3 (75) |
| **Treated with dignity & respect** |  |  |  |  |
| No | 3 (75) | 1 (25) | 1 (25) | 2 (50) |
| Yes | 1 (25) | 3 (75) | 3 (75) | 2 (50) |
| **Had confidence & trust in HCP** |  |  |  |  |
| No | 1 (25) | 1 (25) | 1 (25) | 1 (25) |
| Yes | 3 (75) | 3 (75) | 3 (75) | 3 (75) |
| **Did it ever happen that HCP said one thing & another said something else** |  |  |  |  |
| Never | 2 (50) | 2 (50) | 1 (25) | 2 (50) |
| Only once |  |  | 1 (25) | 2 (50) |
| I don’t know | 2 (50) | 2 (50) | 2 (50) |  |
| **Ever think that the HCPs were deliberately not telling you things** |  |  |  |  |
| No | 3 (75) | 3 (75) | 3 (75) | 3 (75) |
| Yes |  |  |  |  |
| I don’t know | 1 (25) | 1 (25) | 1 (25) | 1 (25) |
| **Receive enough information** |  |  |  |  |
| No | 1 (25) | 1 (25) | 1 (25) | 1 (25) |
| Yes | 3 (75) | 3 (75) | 3 (75) | 3 (75) |
| **When you had questions, did you get answers that you could understand** |  |  |  |  |
| I did not have the opportunity to ask questions | 2 (50) | 2 (50) | 1 (25) | 1 (25) |
| Yes | 2 (50) | 2 (50) | 3 (75) | 3 (75) |
| **Had HCP to contact if you had questions** |  |  |  |  |
| No | 2 (50) | 3 (75) | 3 (75) | 2 (50) |
| Yes | 2 (50) | 1 (25) | 1 (25) | 2 (50) |
| **Had HCP to talk to about worries/fear** |  |  |  |  |
| No | 2 (50) | 2 (50) | 2 (50) | 2 (50) |
| Yes | 2 (50) | 2 (50) | 2 (50) | 2 (50) |
| **Did HCPs do everything they could for your RM care** |  |  |  |  |
| No | 1 (25) | 1 (25) | 1 (25) | 1 (25) |
| Yes | 3 (75) | 3 (75) | 3 (75) | 3 (75) |
|  | | | | |

*RMC, recurrent miscarriage clinic; HCP, healthcare professional*

**Figure S2.3. RM care experience (Men, n=4)**

*The overall experience of RM care (scale from 1-10) recoded as poor (rating 1-3), satisfactory (rating 4-6) and good (rating 7-10)*
