# Supplementary material for: Factors that shape recurrent miscarriage care experiences: findings from a national survey
Source: BMC Health Serv Res. 2023 Mar 31;23:317. doi: 10.1186/s12913-023-09347-1 (PMC10064661; doi:10.1186/s12913-023-09347-1)
Supplement: Supplementary file 3 — Additional file 3: Table S3.1. Women’s characteristics by overall RM care experience rating. Table S3.2. Care received by women during investigations for RM. Table S3.3. Care received by women when receiving the results of their investigations for RM. Table S3.4. Care received by women when getting their treatment plan for RM. Table S3.5. Care received by women for a subsequent pregnancy following RM. Table S3.6. Information and support services used by women for RM. Table S3.7. Women’s overall RM care experience rating. [file 12913_2023_9347_MOESM3_ESM.docx]

**Additional File 3.**

**Table S3.1. Women’s characteristics by overall RM care experience rating, n (%)**

|  | **Overall care experience (n=135)** | | |  |
| --- | --- | --- | --- | --- |
| **Variable (n=135)** | ***Poor (n=60)*** | ***Satisfactory (n=52)*** | ***Good (n=23)*** | ***P-value*** |
| **Age** |  |  |  |  |
| 24-34 years | 15 (65.22) | 7 (30.43) | 1 (4.35) | 0.196 |
| 35-44 years | 43 (40.57) | 42 (39.62) | 21 (19.81) |  |
| 55-64 years | 2 (33.33) | 3 (50.00) | 1 (16.67) |  |
| **Nationality** |  |  |  |  |
| Irish | 57 (44.53) | 48 (37.50) | 23 (17.97) | 0.382 |
| Other | 3 (42.86) | 4 (57.14) | 0 (0.00) |  |
| **Relationship status** |  |  |  |  |
| Married | 50 (43.86) | 45 (39.47) | 19 (16.67) | 0.802 |
| Living with a partner | 7 (43.75) | 5 (31.25) | 4 (25.00) |  |
| Separated or divorced | 1 (100.00) | 0 (0.00) | 0 (0.00) |  |
| Single | 1 (33.33) | 2 (66.67) | 0 (0.00) |  |
| Prefer not to say | 1 (100.00) | (0.00) | 0 (0.00) |  |
| **Education** |  |  |  |  |
| Secondary school or less | 3 (50.00) | 2 (33.33) | 1 (16.7) | 0.231 |
| Post-secondary school technical training | 5 (55.56) | 1 (11.11) | 3 (33.33) |  |
| University degree | 11 (30.56) | 20 (55.56) | 5 (13.89) |  |
| Postgraduate Certificate or Diploma | 17 (54.84) | 11 (35.48) | 3 (9.68) |  |
| Postgraduate Degree (Masters or PhD) | 24 (45.28) | 18 (33.96) | 11 (20.75) |  |
| **Employment** |  |  |  |  |
| Employed full-time | 46 (44.23) | 42 (40.38) | 16 (15.38) | 0.720 |
| Employed part-time | 5 (35.71) | 6 (42.86) | 3 (21.43) |  |
| Self-employed | 2 (40.00) | 2 (40.00) | 1 (20.00) |  |
| Full-time student | 1 (100.00) | 0 (0.00) | 0 (0.00) |  |
| Part-time student | 2 (66.67) | 0 (0.00) | 1 (33.33) |  |
| Not employed | 2 (50.00) | 0 (0.00) | 2 (50.00) |  |
| Prefer not to say | 0 (0.00) | 1 (100.00) | 0 (0.00) |  |
| Other | 2 (66.67) | 1 (33.33) | 0 (0.00) |  |
| **Medical Cover** |  |  |  |  |
| A medical or GP visit card holder | 3 (37.50) | 3 (37.50) | 2 (25.00) | 0.849 |
| Private health insurance holder | 49 (45.37) | 40 (37.04) | 19 (17.59) |  |
| None of the above | 8 (42.11) | 9 (47.37) | 2 (10.53) |  |
| **Consecutive loss (n=135)** |  |  |  |  |
| Two | 33 (42.86) | 33 (42.86) | 11 (14.29) | 0.669 |
| Three | 16 (47.06) | 12 (35.29) | 6 (17.65) |  |
| Four or more | 11 (45.83) | 7 (29.17) | 6 (25.00) |  |
| **Year received RM care** |  |  |  |  |
| 2011-2015 | 15 (44.12) | 11 (32.35) | 8 (23.53) | 0.455 |
| 2016-2021 | 45 (44.55) | 41 (40.59) | 15 (14.85) |  |
| **Diagnosed with infertility (n=135)** |  |  |  |  |
| No | 48 (46.60) | 39 (37.86) | 16 (15.53) | 0.583 |
| Yes | 12 (37.50) | 13 (40.63) | 7 (21.88) |  |

**Tables S3.2-S3.7 present the care received by women at various stages of the RM care pathway**

Abbreviations: HCP, healthcare professional; RM recurrent miscarriage; apt, appointment.

**Table S3.2.** **Care received by women during investigations for RM**

| **Variable** | **N (%)** |
| --- | --- |
| **Investigations** |  |
| **Did a HCP discuss RM with you (n=135)** |  |
| No | 42 (31.11) |
| Yes | 89 (65.93) |
| I don’t know / I can’t remember | 4 (2.96) |
| **After how many miscarriages was RM discussed (n=135)** |  |
| 2 | 53 (59.55) |
| 3 | 29 (32.58) |
| More than 3 | 7 (7.87) |
| **Who was the first HCP to discuss RM with you (n=135)** |  |
| GP | 19 (21.36) |
| Midwife/nurse in the hospital | 16 (17.98) |
| Sonographer | 9 (10.11) |
| Consultant In a public hospital | 15 (16.85) |
| Private consultant | 14 (15.73) |
| HCP infertility | 14 (15.73) |
| Other | 2 (2.25) |
| **Have you had investigations for RM (n=135)** |  |
| No | 64 (47.41) |
| Yes | 71 (52.59) |
| **Were you offered investigations (n=71)** |  |
| I was offered | 27 (38.03) |
| I requested | 44 (61.97) |
| **Wait time for investigation appointment (n=71)** |  |
| Less than 1 month | 20 (28.17) |
| 1-2 months | 14 (19.72) |
| 3-4 months | 25 (35.21) |
| 5-12 months | 8 (11.27) |
| I don’t know/can’t remember | 4 (5.63) |
| **Types of Investigations** |  |
| **Medical history (n=71)** |  |
| No | 16 (22.53) |
| Yes | 55 (77.46) |
| **Blood test (n=71)** |  |
| No | 2 (2.82) |
| Yes | 69 (97.18) |
| **Ultrasound (n=71)** |  |
| No | 11 (15.04) |
| Yes | 60 (84.51) |
| **MRI (n=71)** |  |
| No | 67 (94.37) |
| Yes | 4 (5.63) |
| **Hysteroscopy (n=71)** |  |
| No | 46 (65.71) |
| Yes | 24 (34.29) |
| **Genetic testing pregnancy tissue (n=71)** |  |
| No | 37 (52.11) |
| Yes | 34 (47.89) |
| **Genetic testing (n=71)** |  |
| No | 30 (42.25) |
| Yes | 41 (57.75) |
| **Other investigation (n=71)** |  |
| No | 57 (80.28) |
| Yes | 14 (19.72) |
| **HCP did everything to investigate the cause of RM (n=71)** |  |
| No | 35 (49.30) |
| Yes, definitely | 10 (14.08) |
| Yes, to some extent | 26 (36.62) |

**Table S3.3. Care received by women when receiving the results of their investigations for RM**

| **Variable** | **N (%)** |
| --- | --- |
| **Receiving Results** |  |
| **Received results (n=71)** |  |
| No | 10 (14.08) |
| Yes | 61 (85.92) |
| **Wait time for results (n=61)** |  |
| 0-2 month | 12 (19.67) |
| 3-4 months | 17 (27.87) |
| 5-12 months | 7 (11.49) |
| I don’t know/ can’t remember | 23 (37.70) |
| **How did you feel about the wait time (n=61)** |  |
| About right | 27 (44.26) |
| Too long | 31 (50.82) |
| I don’t know/ I can’t remember | 3 (4.92) |
| **HCP who provided results:** |  |
| **GP (n=61)** |  |
| No | 40 (65.57) |
| Yes | 21 (34.43) |
| **Midwife / nurse at the hospital (n=61)** |  |
| No | 46 (75.41) |
| Yes | 15 (24.59) |
| **Sonographer (n=61)** |  |
| No | 48 (78.69) |
| Yes | 13 (21.31) |
| **Consultant in a public hospital (n=61)** |  |
| No | 41 (67.21) |
| Yes | 20 (32.79) |
| **Doctor in a public hospital (n=61)** |  |
| No | 51 (83.61) |
| Yes | 10 (16.39) |
| **Private consultant (n=61)** |  |
| No | 30 (49.18) |
| Yes | 31 (50.82) |
| **Doctor/midwife/nurse at a fertility clinic (n=61)** |  |
| No | 38 (62.30) |
| Yes | 23 (37.70) |
| **Admin staff (n=61)** |  |
| No | 53 (86.89) |
| Yes | 8 (13.11) |
| **Other (n=61)** |  |
| No | 58 (95.08) |
| Yes | 3 (4.92) |
| **Results received by phone (n=61)** |  |
| No | 30 (49.18) |
| Yes | 31 (50.82) |
| **Results received by email (n=61)** |  |
| No | 50 (81.97) |
| Yes | 11 (18.03) |
| **Results received by letter (n=61)** |  |
| No | 46 (75.41) |
| Yes | 15 (24.59) |
| **Results received face to face (n=61)** |  |
| No | 11 (18.03) |
| Yes | 50 (81.97) |
| **Results received by virtual communication (n=61)** |  |
| No | 57 (93.44) |
| Yes | 4 (6.56) |
| **Results received by another method (n=61)** |  |
| No | 56 (91.80) |
| Yes | 5 (8.20) |
| **Did the results provide answers for your RM (n=61)** |  |
| No | 39 (63.93) |
| Yes | 21 (34.43) |
| I don’t know | 1 (1.64) |

**Table S3.4. Care received by women when getting their treatment plan for RM**

| **Variable** | **N (%)** |
| --- | --- |
| **Treatment/ Care plan** |  |
| **Was a treatment plan put in place (n=135)** |  |
| No | 62 (45.93) |
| Yes | 70 (51.85) |
| **HCP who provided the treatment plan (n=70)**  **GP** |  |
| No | 57 (81.43) |
| Yes | 13 (18.57) |
| **Midwife/nurse at the hospital (n=70)** |  |
| No | 60 (85.71) |
| Yes | 10 (14.29) |
| **Sonographer (n=70)** |  |
| No | 68 (97.14) |
| Yes | 2 (2.86) |
| **Consultant in a public hospital (n=70)** |  |
| No | 58 (82.86) |
| Yes | 12 (17.14) |
| **Doctor in a public hospital (n=70)** |  |
| No | 62 (88.57) |
| Yes | 8 (11.43) |
| **Private consultant (n=70)** |  |
| No | 39 (55.71) |
| Yes | 31 (44.29) |
| **Doctor/midwife/nurse at a fertility clinic (n=70)** |  |
| No | 40 (57.14) |
| Yes | 30 (42.86) |
| **Other (n=70)** |  |
| No | 66 (94.29) |
| Yes | 4 (5.71) |
| **HCP did everything they could to treat RM (n=135)** |  |
| No | 23 (41.49) |
| Yes definitely | 21 (30.00) |
| Yes, to some extent | 20 (28.57) |

**Table S3.5. Care received by women for a subsequent pregnancy following RM**

| **Variable** | **N (%)** |
| --- | --- |
| **Subsequent pregnancy** |  |
| **Experienced another pregnancy (n=135)** |  |
| No | 38 (28.15) |
| Yes | 97 (71.85) |
| **Offered early reassurance scans (n=97)** |  |
| No | 21 (21.65) |
| Yes | 76 (78.25) |
| **Where they received subsequent pregnancy care**  **General practice (n=97)** |  |
| No | 38 (39.16) |
| Yes | 59 (60.82) |
| **Hospital (n=97)** |  |
| No | 69 (71.13) |
| Yes | 28 (28.87) |
| **Hospital pregnancy loss clinic (n=97)** |  |
| No | 93 (95.88) |
| Yes | 4 (4.12) |
| **Hospital recurrent miscarriage clinic (n=97)** |  |
| No | 91 (93.81) |
| Yes | 6 (6.19) |
| **Hospital early pregnancy assessment unit (n=97)** |  |
| No | 37 (38.14) |
| Yes | 60 (61.86) |
| **Hospital gynaecology clinic (n=97)** |  |
| No | 91 (93.81) |
| Yes | 6 (6.19) |
| **Private consultant rooms (n=97)** |  |
| No | 57 (58.76) |
| Yes | 40 (41.24) |
| **Fertility clinic (n=97)** |  |
| No | 77 (79.28) |
| Yes | 20 (20.62) |
| **Other (n=97)** |  |
| No | 90 (92.78) |
| Yes | 7 (7.22) |
| **HCPs did everything to support them during their subsequent pregnancy (n=97)** |  |
| No | 29 (29.90) |
| Yes, definitely | 29 (29.90) |
| Yes, to some extent | 38 (39.18) |
| I don’t know | 1 (1.03) |

**Table S3.6. Information and support services used by women for RM**

| **Information & support services used (n=135)** | | | |
| --- | --- | --- | --- |
|  | *No* | *Yes* | *I don’t know/ can’t remember* |
| **Specialist in bereavement & loss** | 71 (52.59) | 60 (44.44) | 4 (2.96) |
| **Miscarriage Association of Ireland** | 68 (50.37) | 62 (45.93) | 3 (3.70) |
| **Féileacáin** | 103 (76.30) | 26 (19.26) | 6 (4.44) |
| **Online discussion boards/forums** | 116 (88.55) | 13 (9.92) | 2 (1.53) |
| **Chaplaincy and/or pastoral care** | 115 (85.19) | 15 (11.11) | 5 (3.70) |
| **Religious or spiritual support group** | 125 (92.59) | 6 (4.44) | 4 (2.96) |
| **One-on-one counselling** | 113 (83.70) | 19 (14.07) | 3 (2.22) |
| **Psychiatry** | 129 (95.56) | 2 (1.48) | 4 (2.96) |
| **Social work service** | 129 (96.27) | 2 (1.49) | 3 (2.24) |
| **Perinatal mental health** | 128 (94.81) | 4 (2.96) | 3 (2.22) |
| **Books/booklets/leaflets/pamphlets** | 51 (37.78) | 80 (59.26) | 4 (2.96) |
| **Pregnancy and Infant Loss website** | 100 (74.07) | 23 (17.04) | 12 (8.89) |
| **Cork Miscarriage website** | 112 (82.96) | 7 (5.19) | 7 (5.19) |

**Table S3.7. Women’s overall RM care experience rating**

| **Variable** | **N (%)** |
| --- | --- |
| **Overall care (n=135)** |  |
| **Rating of overall RM care experience*** |  |
| Poor | 60 (44.44) |
| Satisfactory | 52 (38.52) |
| Good | 23 (17.04) |
| **Staff in different places worked well together** |  |
| No | 81 (60.00) |
| Yes | 54 (40.00) |
| **Expectation of care** |  |
| Much better than expected | 8 (5.93) |
| Somewhat better than expected | 13 (9.63) |
| As expected | 27 (20.00) |
| Somewhat worse than expected | 39 (28.89) |
| Much worse than expected | 48 (35.56) |

***** *The overall experience of RM care (scale from 1-10) recoded as poor (rating 1-3), satisfactory (rating 4-6) and good (rating 7-10)*

## 
