# Supplementary material for: Factors that shape recurrent miscarriage care experiences: findings from a national survey
Source: BMC Health Serv Res. 2023 Mar 31;23:317. doi: 10.1186/s12913-023-09347-1 (PMC10064661; doi:10.1186/s12913-023-09347-1)
Supplement: Supplementary file 4 — Additional file 4: Table S4.1. Unadjusted associations for patient-centred care items during investigations by overall care experience rating. Table S4.2. Unadjusted associations for patient-centred care items when receiving results by overall care experience rating. Table S4.3. Unadjusted associations for patient-centred care items for treatment/plan of care by overall care experience rating. Table S4.4. Unadjusted associations for patient-centred care items during subsequent pregnancy care by overall care experience rating. [file 12913_2023_9347_MOESM4_ESM.docx]

**Additional File 4.**

## Tables S4.1-S4.4 present finds from the unadjusted multinomial logistic regression analysis

Abbreviations: HCP, healthcare professional; RM recurrent miscarriage; apt, appointment.

## Table S4.1. Unadjusted associations for patient-centred care items during investigations by overall care experience rating

| **Variable** | **Overall RM care experience** | | | | |
| --- | --- | --- | --- | --- | --- |
| **Investigations** | **Satisfactory ^a^ (n =52)** |  | **Good^a^ (n=23)** | |  |
| No | 1 |  | 1 | |  |
| Yes | 0.81 (0.39-1.70) | 0.579 | 1.36 (0.51-3.62) | | 0.537 |
| **Offered investigations** |  |  |  | |  |
| I was offered investigations | 1 |  | 1 | |  |
| I requested investigations | 0.5 (0.16-1.55) | 0.230 | 0.19 (0.48-0.712) | | 0.015 |
| **Enough time to discuss investigations** |  |  |  | |  |
| No | 1 |  | 1 | |  |
| Yes | 3.25 (1.06-0.97) | 0.039 | 11 (2.44-49.53) | | 0.002 |
| **Involved as much as you wanted to be in decisions** |  |  |  | |  |
| No | 1 |  | 1 | |  |
| Yes | 2.6 (0.88-7.64) | 0.083 | 19 (2.21-163.57) | | 0.007 |
| **Treated with dignity & respect** |  |  |  | |  |
| No | 1 |  | 1 | |  |
| Yes | 3.83 (0.73-19.99) | 0.111 | 0 (0-0) | | 0.991 |
| **Had confidence & trust in HCP** |  |  |  | |  |
| No | 1 |  | 1 | |  |
| Yes | 5.23 (1.03-26.62) | 0.046 | 92 (0-0) | | 0.989 |
| **Did it ever happen that HCP said one thing & another said something else** |  |  |  | |  |
| Never | 1 |  | 1 | |  |
| Often | 0.13 (0.02-0.63) | 0.012 | 0.06 (0.01-0.72) | | 0.027 |
| Sometimes | 0.54 (0.13-2.27) | 0.402 | 0.75 (0.15-3.84) | | 0.730 |
| **Ever think that the HCPs were deliberately not telling you things** |  |  |  | |  |
| No | 1 |  | 1 | |  |
| Yes | 0.31 (0.12-0.96) | 0.043 | 0.38 (0.10-1.40) | | 0.145 |
| **Received enough info on investigations** |  |  |  | |  |
| No | 1 |  | 1 | |  |
| Yes | 2.81 (0.89-8.88) | 0.079 | 13.10 (2.84-60.30) | | 0.001 |
| **Received written info** |  |  |  | |  |
| No | 1 |  | 1 | |  |
| Yes | 3.05 (0.68-13.71) | 0.145 | 5.37 (1.06-27.00) | | 0.041 |
| **When you had questions about investigations, did you get answers that you could understand** |  |  |  | |  |
| No | 1 |  | 1 | |  |
| Yes | 2.17 (0.68-6.90) | 0.191 | 8.89 (1.03-76.58) | | 0.047 |
| **Told/given info about who to contact while waiting for results** |  |  |  |  | |
| No | 1 |  | 1 | |  |
| Yes | 1.23 (0.43-3.50) | 0.701 | 4.16 (0.97-17.77) | | 0.055 |
| **Had HCP to talk to about worries/fear** |  |  |  | |  |
| No | 1 |  | 1 | |  |
| Yes | 3.54 (1.17-10.68) | 0.025 | 6.11 (1.41-26.41) | | 0.015 |
| **Rating of investigation waiting area** |  |  |  | |  |
| Good | 1 |  | 1 | |  |
| Satisfactory | 0.62 (0.16-2.42) | 0.493 | 0.7 (0.16-3.10) | | 0.638 |
| Poor | 0.41 (0.11-1.53) | 0.187 | 0.16 (0.02-0.97) | | 0.047 |
| **Rating of investigation consultation room** |  |  |  | |  |
| Good | 1 |  | 1 | |  |
| Satisfactory | 1.18 (0.38-3.67) | 0.773 | 9.11 (0-0) | | 0.995 |
| Poor | 6.38 (0-0) | 0.996 | 0.10 (0.01-0.93) | | 0.043 |
| **Someone attended the investigation apt with you** |  |  |  | |  |
| No | 1 |  | 1 | |  |
| Yes | 0.60 (0.16-2.29) | 0.455 | 1.10 (0.23-5.28) | | 0.905 |
| Not facilitated due to covid | 1.17 (0.24-5.62) | 0.847 | 4.9 (0-0) | | 0.986 |
| **Partner able to ask HCP questions** |  |  |  | |  |
| No | 1 |  | 1 | |  |
| Yes | 1.18 (0.36-3.90) | 0.781 | 3.83 (1.01-14.48) | | 0.048 |
| I do not have a partner | 1.35 (0.08-23.19) | 0.835 | 4.15 (0-0) | | 0.990 |
| **Did HCPs do everything investigate your RM** |  |  |  | |  |
| No | 1 |  | 1 | |  |
| Yes | 7.59 (2.32-24.87) | 0.001 | 21.43 (3.85-119.14) | | 0.000 |
| ^a^Reference category: poor experience of RM care; ^d^1 denotes reference category | | | | | |

##

## Table S4.2. Unadjusted associations for patient-centred care items when receiving results by overall care experience rating

| **Variable** | **RM care experience** | | | |
| --- | --- | --- | --- | --- |
| **Received results of investigation** | **Satisfactory ^a^ (n =52)** |  | **Good^a^ (n=23)** |  |
| No | 1 |  | 1 |  |
| Yes | 1.36 (0.29-6.32) | 0.697 | 1.11 (0.19-6.56) | 0.907 |
| **Enough time to discuss your results** |  |  |  |  |
| No | 1 |  | 1 |  |
| Yes | 3.64 (1.10-11.97) | 0.033 | 3.9 (0-0) | 0.990 |
| **Treated with dignity & respect** |  |  |  |  |
| No | 1 |  | 1 |  |
| Yes | 2.22 (0.50-9.85) | 0.295 | 637 (0-0) | 0.990 |
| **Had confidence & trust in HCP** |  |  |  |  |
| No | 1 |  | 1 |  |
| Yes | 1.89 (0.49-7.40) | 0.358 | 4.63 (0.51-42.11) | 0.174 |
| **Did it ever happen that HCP said one thing & another said something else** |  |  |  |  |
| Never | 1 |  | 1 |  |
| Often | 0.14 (0.01-1.44) | 0.099 | 0.17 (0.02-1.72) | 0.132 |
| Sometimes | 1.45 (0.40-5.26) | 0.568 | 0.61 (0.14-2.71) | 0.512 |
| **Ever think that the HCPs were deliberately not telling you things** |  |  |  |  |
| No | 1 |  | 1 |  |
| Yes | 0.24 (0.06-0.90) | 0.034 | 0.54 (0.13-2.22) | 0.392 |
| **Received enough info on results** |  |  |  |  |
| No | 1 |  | 1 |  |
| Yes | 1.99 (0.62-6.43) | 0.250 | 10.21 (1.15-90.53) | 0.037 |
| **Received written info results** |  |  |  |  |
| No | 1 |  | 1 |  |
| Yes | 1.33 (0.38-4.62) | 0.650 | 1.43 (0.33-6.26) | 0.636 |
| **When you had questions about results, did you get answers that you could understand** |  |  |  |  |
| No | 1 |  | 1 |  |
| Yes | 2.65 (0.70-10.7) | 0.153 | 2.94 (0.53-16.22) | 0.216 |
| **Told/given info about who to contact with questions about your result** |  |  |  |  |
| No | 1 |  | 1 |  |
| Yes | 1.38 (0.43-4.45) | 0.585 | 2.80 (0.69-11.34) | 0.149 |
| **HCP talk to about worries/fear** |  |  |  |  |
| No | 1 |  | 1 |  |
| Yes | 2.98 (0.93-9.57) | 0.067 | 18.70 (2.09-167.27) | 0.009 |
| **Rating of results waiting area** |  |  |  |  |
| Good | 1 |  | 1 |  |
| Satisfactory | 0.75 (0.16-3.53) | 0.716 | 0.5 (0.10-2.60) | 0.410 |
| Poor | 0.77 (0.19-3.12) | 0.713 | 0.15 (0.02-1.00) | 0.050 |
| **Rating of results consultation room** |  |  |  |  |
| Good | 1 |  | 1 |  |
| Satisfactory | 0.70 (0.18-2.66) | 0.601 | 8.07 (0-0) | 0.987 |
| Poor | 0.75 (0.18-3.06) | 0.688 | 0.10 (0.01-0.98) | 0.048 |
| **Someone attended the result apt with you** |  |  |  |  |
| No | 1 |  | 1 |  |
| Yes | 1.89 (0.53-6.69) | 0.326 | 2.1 (0.47-9.30) | 0.328 |
| Not facilitated due to Covid-19 restrictions | 1.37 (0.27-6.87) | 0.701 | 0.6 (0.05-6.79) | 0.680 |
| **Partner able to ask HCP questions** |  |  |  |  |
| No | 1 |  | 1 |  |
| Yes | 1.31 (0.32-5.32) | 0.703 | 5.88 (1.30-26.51) | 0.021 |
| I do not have a partner | 1.31 (0.08-22.63) | 0.851 | 1.5 (0-0) | 0.994 |
| **Results provided an answer for your RM** |  |  |  |  |
| No | 1 |  | 1 |  |
| Yes | 0.55 (0.16-1.83) | 0.327 | 0.73 (0.17-3.02) | 0.661 |
| ^a^Reference category: poor experience of RM care; ^d^1 denotes reference category | | | | |

## Table S4.3. Unadjusted associations for patient-centred care items for treatment/plan of care by overall care experience rating

| **Variable** | **RM care experience** | | | |
| --- | --- | --- | --- | --- |
| **Treatment plan received** | **Satisfactory ^a^ (n =52)** |  | **Good^a^ (n=23)** |  |
| No | 1 |  | 1 |  |
| Yes | 1.41 (0.67-2.98) | 0.364 | 3.71 (1.28-10.71) | 0.016 |
| **Enough time to discuss your treatment plan** |  |  |  |  |
| No | 1 |  | 1 |  |
| Yes | 2.33 (0.66-8.24) | 0.190 | 73 (0-0) | 0.986 |
| **Involved as much as you wanted to be in decisions regarding treatment** |  |  |  |  |
| No | 1 |  | 1 |  |
| Yes | 2.4 (0.53-10.84) | 0.255 | 2.25 (0.40-2.00) | 0.360 |
| **Treated with dignity & respect** |  |  |  |  |
| No | 1 |  | 1 |  |
| Yes | 2.27 (0.38-13.64) | 0.369 | 416 (0-0) | 0.990 |
| **Had confidence & trust in HCP** |  |  |  |  |
| No | 1 |  | 1 |  |
| Yes | 3.75 (0.68-20.62) | 0.129 | 2.3 (0-0) | 0.994 |
| **Did it ever happen that HCP said one thing & another said something else** |  |  |  |  |
| Never | 1 |  | 1 |  |
| Often | 0.3 (0.06-1.47) | 0.137 | 0.1 (0.01-0.95) | 0.045 |
| Sometimes | 1.58 (0.45-5.50) | 0.477 | 0.68 (0.17-2.71) | 0.579 |
| **Ever think that the HCPs were deliberately not telling you things** |  |  |  |  |
| No | 1 |  | 1 |  |
| Yes | 0.95 (0.28-3.22) | 0.934 | 0.36 (0.07-2.00) | 0.244 |
| **Received enough info about treatment plan** |  |  |  |  |
| No | 1 |  | 1 |  |
| Yes | 3.04 (0.80-11.55) | 0.102 | 846 (0-0) | 0.987 |
| **Received written info treatment plan** |  |  |  |  |
| No | 1 |  | 1 |  |
| Yes | 2.1 (0.59-7.41) | 0.249 | 1.75 (0.42-7.30) | 0.442 |
| **When you had questions, did you get answers that you could understand** |  |  |  |  |
| No | 1 |  | 1 |  |
| Yes | 1.90 (0.41-8.94) | 0.414 | 3.81 (0.40-35.91) | 0.243 |
| **Told/given info about who to contact if questions regarding your treatment** |  |  |  |  |
| No | 1 |  | 1 |  |
| Yes | 2.73 (0.89-8.33) | 0.078 | 6.36 (1.46-27.67) | 0.014 |
| **HCP to talk to about worries/fear** |  |  |  |  |
| No | 1 |  | 1 |  |
| Yes | 5.6 (1.68-18.65) | 0.005 | 12 (2.25-63.98) | 0.004 |
| **Partner able to ask HCP questions** |  |  |  |  |
| No | 1 |  | 1 |  |
| Yes | 1.78 (0.49-6.43) | 0.381 | 4.50 (1.15-17.65) | 0.031 |
| I do not have a partner | 1.11 (0.06-19.09) | 0.942 | 8.2 (0-0) | 0.994 |
| **HCP did everything to treat your RM** |  |  |  |  |
| No | 1 |  | 1 |  |
| Yes | 3.83 (1.22-11.98) | 0.021 | 36 (4.05-320.12) | 0.001 |
| ^a^Reference category: poor experience of RM care; ^d^1 denotes reference category | | | | |

##

## Table S4.4. Unadjusted associations for patient-centred care items during subsequent pregnancy care by overall care experience rating

| **Variable** | **RM care experience** | | | |
| --- | --- | --- | --- | --- |
| **Had a subsequent pregnancy** | **Satisfactory (n =52)** |  | **Good^a^ (n=23)** |  |
| No | 1 |  | 1 |  |
| Yes | 2.00 (0.86-4.70) | 0.109 | 1.53 (0.52-4.45) | 0.440 |
| **Offered reassurance scans** |  |  |  |  |
| No | 1 |  | 1 |  |
| Yes | 3.27 (1.10-9.67) | 0.033 | 8.96 (1.07-74.91) | 0.043 |
| **Enough time to discuss concerns pregnancy** |  |  |  |  |
| No | 1 |  | 1 |  |
| Yes | 5.67 (2.03-15.85) | 0.001 | 8.75 (1.76-43.52) | 0.008 |
| **Involved as much as you wanted to be in decisions regarding your pregnancy** |  |  |  |  |
| No | 1 |  | 1 |  |
| Yes | 4.62 (1.35-15.78) | 0.015 | 852 (0-0) | 0.987 |
| **Treated with dignity & respect** |  |  |  |  |
| No | 1 |  | 1 |  |
| Yes | 1.9 (0-0) | 0.989 | 1.9 (0-0) | 0.993 |
| **Had confidence & trust in HCP** |  |  |  |  |
| No | 1 |  | 1 |  |
| Yes | 5.85 (1.18-29.14) | 0.031 | 28(0-0) | 0.984 |
| **Did it ever happen that HCP said one thing & another said something else** |  |  |  |  |
| Never | 1 |  | 1 |  |
| Often | 2.24 (0-0) | 0.990 | 1.75 (0-0) | 0.994 |
| Sometimes | 0.88 (0.32-2.41) | 0.807 | 0.56 (0.16-1.92) | 0.352 |
| **Ever think that the HCPs were deliberately not telling you things** |  |  |  |  |
| No | 1 |  | 1 |  |
| Yes | 0.20 (0.07-0.55) | 0.002 | 0.20 (0.05-0.82) | 0.025 |
| **When you had questions, did you get answers that you could understand regarding your pregnancy** |  |  |  |  |
| No | 1 |  | 1 |  |
| Yes | 2.06 (0.74-5.72) | 0.164 | 8 (0.95-67.13) | 0.055 |
| **Told/given info about who to contact if questions regarding your pregnancy** |  |  |  |  |
| No | 1 |  | 1 |  |
| Yes | 2.04 (0.84-5.00) | 0.116 | 12.00 (2.40-60.05) | 0.002 |
| **Had HCP to talk to about worries/fear** |  |  |  |  |
| No | 1 |  | 1 |  |
| Yes | 1.65 (0.67-4.07) | 0.275 | 6.43 (1.29-32.0) | 0.023 |
| **Rating of waiting area for subsequent pregnancy care** |  |  |  |  |
| Good | 1 |  | 1 |  |
| Satisfactory | 0.77 (0.24-2.52) | 0.668 | 0.38 (0.95-1.49) | 0.163 |
| Poor | 0.48 (0.17-1.41) | 0.182 | 4.06 (0-0) | 0.986 |
| **Rating of consultation room for subsequent pregnancy care** |  |  |  |  |
| Good | 1 |  | 1 |  |
| Satisfactory | 0.85 (0.31-2.32) | 0.749 | 0.09 (0.01-0.77) | 0.028 |
| Poor | 0.33 (0.10-1.14) | 0.080 | 0.10 (0.01-0.84) | 0.035 |
| **Someone attended a pregnancy apt with you** |  |  |  |  |
| No | 1 |  | 1 |  |
| Yes | 1.45 (0.38-5.51) | 0.583 | 3.79 (0.40-35.19) | 0.243 |
| Not facilitated due to Covid | 1.11 (0.27-4.55) | 0.880 | 1.71 (0.16-18.73) | 0.659 |
| **Partner able to ask HCP questions** |  |  |  |  |
| No | 1 |  | 1 |  |
| Yes | 0.94 (0.31-2.81) | 0.908 | 2.53 (0.76-9.08) | 0.128 |
| I do not have a partner | 0.94 (0.06-15.67) | 0.964 | 2.67 (0-0) | 0.990 |
| **HCP did everything to support your subsequent pregnancy** |  |  |  |  |
| No | 1 |  | 1 |  |
| Yes | 4.15 (1.57-10.95) | 0.004 | 102 (0-0) | 0.982 |
| ^a^Reference category: poor experience of RM care; ^d^1 denotes reference category | | | | |
